# Practical Considerations of PRN Medicines Management: An Integrative Systematic Review

**DOI:** 10.3389/fphar.2022.759998

**Published:** 2022-04-12

**Authors:** Abbas Mardani, Piret Paal, Christiane Weck, Shazia Jamshed, Mojtaba Vaismoradi

**Affiliations:** ^1^ Nursing Care Research Center, Department of Medical Surgical Nursing, School of Nursing and Midwifery, Iran University of Medical Sciences, Tehran, Iran; ^2^ Institute of Nursing Science and Practice, Paracelsus Medical University, Salzburg, Austria; ^3^ Palliative Care, Paracelsus Medical University, Salzburg, Austria; ^4^ Department of Neurology, Klinikum Agatharied, Hausham, Germany; ^5^ Clinical Pharmacy and Practice, Faculty of Pharmacy, University Sultan Zainal Abidin, Terengganu, Malaysia; ^6^ Faculty of Nursing and Health Sciences, Nord University, Bodø, Norway

**Keywords:** clinical practice, medication, medicines management, patient safety, pro re nata

## Abstract

**Background and objectives:** Highly widespread use of *pro re nata* (PRN) medicines in various healthcare settings is a potential area for improper medication prescription and administration leading to patient harm. This study aimed to summarize and integrate the findings of all relevant individual studies regarding the practical considerations of PRN medicines management including strategies and interventions by healthcare professionals for safe prescription, dispensing, administration, monitoring, and deprescription of PRN medicines in healthcare settings.

**Methods:** An integrative systematic review on international databases were performed. Electronic databases including Web of Knowledge, Scopus, PubMed (including MEDLINE), and Cinahl were searched to retrieve articles published until end of May 2021. Original qualitative, quantitative, and mixed methods studies written in English were included with a focus on PRN medicines management in healthcare settings. Research synthesis using the narrative method was performed to summarise the results of included studies.

**Results:** Thirty-one studies on PRN medicines in healthcare settings by different healthcare providers were included after the screening of the databases based on eligibility criteria. They were published from 1987 to 2021. The majority of studies were from Australia, the United States, Canada, and the United Kingdom and were conducted in psychiatric settings. Given variations in their purposes, methods, and outcomes, the research synthesis was conducted narratively based on diversities and similarities in findings. Eight categories were developed by the authors as follows: “PRN indications and precautionary measures,” “requirements of PRN prescription,” “interventions for PRN administration,” “monitoring and follow up interventions,” “deprescription strategies,” “healthcare professionals’ role,” “participation of patients and families,” and “multidisciplinary collaboration.” Each category consists of several items and describes what factors should be considered by healthcare professionals for PRN medicines management.

**Conclusion:** The review findings provide insights on the practical considerations of PRN medicines management in clinical practice. The suggested list of considerations in our review can be used by healthcare professionals for optimal PRN medicines management and safeguarding patient care.

## 1 Introduction

Medication therapy is the most common therapeutic intervention (World Health Organization, 2019). Medicines management is the process of the evaluation of the patient’s health status and the need for medications’ prescription and dispensing, and the administration and monitoring of medication’s effectiveness (Car et al., 2017; Mishore et al., 2020). Medication errors are significantly prevalent and happen in up to 67% of patients during hospitalization (Nguyen et al., 2017). They are major international contributors to healthcare complications and the increased costs of healthcare (Acheampong et al., 2014). Therefore, the safe use of medications has a top priority for healthcare systems with an calculated annual burden of $ 42 billion worldwide (World Health Organization, 2018).

Given the frequency and potential association of preventable medication errors with adverse patient outcomes, the development of strategies through medicines management for the reduction of their clinical magnitude are common (Kwan et al., 2013; Basey et al., 2014). Prevention and reduction of medication errors are the primary goals of healthcare organizations through participation in quality improvement initiatives. They are also intertwined with ethical healthcare (Smith, 2013; Pitkänen et al., 2016).

### 1.1 *Pro re nata* Medicines Management in Healthcare Settings

“*Pro re nata*” (PRN), “when required,” or “as needed” is defined as the prescription and administration of medications based on the immediate patients’ needs instead of prescheduled administration times (Martin et al., 2017). PRN medications often are used for relieving symptoms rather than treating patients’ underlying diseases (Harper et al., 2017). Common medications used as PRN are psycholeptic and psychotropic medications including antipsychotics, anxiolytics, sedatives and hypnotics; painkillers; gastrointestinal medications; and other drugs used for mitigating physical and psychological symptoms (Allers et al., 2017; Dörks et al., 2019; Vaismoradi et al., 2020).

PRN medications are prescribed and administered at least once to 68–83.9% of patients suffering from mental health issues (Vaismoradi et al., 2018). In mental healthcare settings, PRN prescriptions have major contributions to the frequency of dangerous high and combined doses of antipsychotic medications that patients receive (Baker et al., 2008). It has been reported that 62–97% of patients treated in mental health wards receive PRN medications especially antipsychotics and psychotropics (Baker et al., 2008; Fujita et al., 2013; Martin et al., 2017; Hipp et al., 2020; Saito et al., 2020). The use of psychotropic medications as PRN is associated with abuse, polypharmacy, increased risks of morbidity, dependency, and risk of falls, which complicate its safety (Hilton and Whiteford, 2008; Nyborg et al., 2017). Therefore, the potential of patient harm should be carefully evaluated (Stroup and Gray, 2018).

This is the nurse responsibility to administer PRN medications based on the patient health condition after receiving the physician’s prescription order (Dörks et al., 2019). Although PRN medications can improve care efficiency, they are accompanied with potential medication safety issues (Oh et al., 2014). Medication errors can happen during the prescription, dispensing, and administration of PRN medications (Vaismoradi et al., 2019). In the intensive care unit, medication errors have been reported in 89% of PRN medication orders (Alaqqad et al., 2016). Improper prescription and administration of PRN medications can cause medication interactions, adverse drug reactions (ADRs), overuse and abuse (Davies et al., 2007; Vaismoradi et al., 2018). PRN medications increase the complexity of medication regimens (Picton et al., 2021). Frequent administration of PRN medications can hide the signs and symptoms of underlying diseases (Vaismoradi et al., 2018).

PRN is considered an unsafe mechanism for medication delivery because the chain of accountability between the decision to prescribe PRN medications and the decision to administer them is unclear (Price and Baker, 2013). The safety of PRN medicines management is influenced by healthcare professionals’ knowledge and skills, and the healthcare culture (Morkunas et al., 2016). The decision for the use of PRN medications are taken in collaboration with the physician, nurse, patient, and families, but it is accompanied by the risk of errors due to their distinct interpretations of the medication process (Hogan et al., 2019). Ambiguities in PRN medicines management including indication for prescription, method of administration, and complete documentation can adversely impact patient care outcomes, increase the risk of polypharmacy, adverse drug events, and abuse (Friedman et al., 2012; Oh et al., 2014; Hammer et al., 2019). The decision for the administration of PRN medications is a nearly independent component of the nursing role after prescribing, and nurses require a clear articulation in clinical practice associated with PRN medication administration (Molloy et al., 2012).

### 1.2 Significance of Understanding the Practical Considerations of PRN Medicines Management

Discrepancies in medicines management between healthcare professionals in various healthcare settings indicate the potential concerns for the use of PRN medications (Stubbings et al., 2019). Nurses often interpret PRN orders for painkillers to be the least amount of PRN medication use. Also, the practice of PRN medications has setting-specific characteristics rather than being evidenced-based (Lellan, 1997; Sonntag et al., 2006). Moreover, there are disparities in the perspectives of healthcare professions especially nurses with regard to the appropriate indications for PRN medications use in patients with different health conditions (Baker et al., 2007b). Furthermore, the monitoring of PRN medications by nurses after administration and related documentation are not properly performed (Friedman et al., 2012; Ross et al., 2021). Making decisions on the use of PRN medications usually is not guideline-based and rather is based on habits and previous experiences in clinical practice (Douglas-Hall and Whicher, 2015; Walsh et al., 2021).

Due to the highly widespread use of PRN medications in various healthcare settings, and the growing concern regarding the use of PRN medications as the first-line choice for relieving patient’s suffering, there is a need for the introduction of evidenced-based protocols and procedures with regard to prescription, dispensing, administration, and monitoring of PRN medications. Also, reviews on PRN medications in terms of indication, frequency, and interdisciplinary collaborations for PRN medicines management is insufficient (Martin et al., 2017; Vaismoradi et al., 2021a). Therefore, this study aimed to summarize and integrate the findings of all relevant individual studies regarding the practical considerations of PRN medicines management including strategies and interventions by healthcare professionals for prescription, dispensing, administration, monitoring, and deprescription of PRN medicines in healthcare settings. The review question was as follows: What are the practical considerations in terms of strategies and interventions by healthcare professionals including nurses, pharmacists and physicians for PRN medicines management in short-term, long-term and acute healthcare settings?

## 2 Materials and Methods

### 2.1 Design

An integrative systematic review was conducted. This is a review method that allows for the inclusion of studies with qualitative and quantitative methodologies and considers a narrative approach for the synthesis of data from a wide range of research designs (Whittemore and Knafl, 2005; Souza et al., 2010). The review protocol was developed by the authors prior to the study, and all steps of the review were conducted accordingly (Supplementary File S1). In addition, PROSPERO was searched to identify ongoing or recently completed similar systematic reviews.

The review question was framed using the PICO statement as follows:

P (Population): healthcare providers including nurses, pharmacists, and physicians who are involved in PRN medicines management; I (Interest): practical considerations in terms of interventions and strategies by healthcare professionals for prescription, dispensing, administration, monitoring, and deprescription of PRN medicines; Co. (Context): all contexts in healthcare consisting of child, adult, physical and mental health.

The review process was informed by the Preferred Reporting Items Systematic Reviews and Meta-analysis (PRISMA) statement (Liberati et al., 2009) (Supplementary File S2).

### 2.2 Search Process

Search keywords and phrases were determined by the research team consisting of the nurse (AM, PP, MV), physician (CW), and pharmacist (SJ) through the review of relevant literature and based on a pilot search in general and specialized databases. The Boolean search method was used with the inclusion of the following keywords:

(PRN OR “pro re nata” OR “as needed” OR “as required”) AND (guideline OR “practice guideline” OR “clinical practice guideline” OR “clinical guideline” OR “critical pathway” OR “clinical pathway” OR “critical path” OR “clinical path” OR “patient care planning” OR instruction OR technique OR program*) AND (medication OR drug OR medicines OR “pharmaceutical preparations” OR pharmaceuticals OR “medicines management”).

The online databases of Web of Knowledge, PubMed (including MEDLINE), Cinahl, and Scopus were searched to retrieve empirical studies published by peer-reviewed scientific journals up to end of May 2021. In addition, cross-references from bibliographies and manual search in the references lists of selected studies were performed to expand the search coverage.

Inclusion criteria for the selection of studies to our review were: qualitative, quantitative, and mixed methods studies with a focus on PRN medicines management; use of any practical consideration in terms of interventions and strategies by healthcare professionals for prescription, administration, monitoring, and management of the side effects and ADRs of PRN medications; published in peer-reviewed scientific journals.

Studies without exact relevance to PRN medicines management were excluded. Also, exclusion encompassed non-empirical studies such as reviews, letters, commentaries, conference proceedings, theses, dissertations, books, and governmental documents that did not provide appropriate data to our review.

The phases of review were carried out separately by two review authors (AM, MV). They shared results and conducted online conversations to make decisions on the subsequent search steps. The studies’ titles, abstracts and full-texts were screened step by step by them. The review authors held discussions in case of discrepancies in their perspectives to reach agreement, and also sought the perspective of the third review author.

### 2.3 Quality Appraisal and Risk of Bias Assessment

Quality of selected studies was evaluated in terms of the appropriateness of research structure and reporting using the Enhancing the Quality and Transparency of Health Research (EQUATOR). According to the studies’ designs, the following tools were used: 1) the Strengthening the Reporting of Observational Studies in Epidemiology (STROBE) for observational and cross-sectional studies; 2) the Standards for Reporting Qualitative Research (SRQR) for qualitative research; 3) Consolidated Standards of Reporting Trials (CONSORT) for experimental and quasi-experimental studies; 4) the Good Reporting of A Mixed Methods Study (GRAMMS) for Mixed-methods studies (EQUATOR Network, 2019).

For making the final decision on whether or not to include studies in the research synthesis, the authors considered scores obtained by the quality appraisal tools and their collective opinions with regard to the significance and the methodological quality of each study.

The Cochrane Collaboration’s tool for assessing the risk of bias for randomized clinical trials was used and the review authors classified their judgments as low, high, and unclear risk of bias (Higgins and Altman, 2011). The Risk of Bias in Non-randomized Studies of Interventions (ROBINS-I) tool was also used along with the categorization of judgments as follows: low, moderate, serious, critical, and no information regarding risk of bias (Sterne et al., 2016). The risk of bias assessment for cross-sectional studies was adapted from the Newcastle-Ottawa Quality Assessment Scale with the judgment’s classification of low, probably low, probably high, and high risk of bias (Herzog et al., 2013).

### 2.4 Data Extraction and Knowledge Synthesis

For data extraction, a table was developed comprising the following sections: 1) the first author’s surname, publication year, and the country where the study was conducted; 2) study design, sample size, and setting; 3) data relating to the practical considerations of PRN medicines management; 4) name and dose of PRN medications and patients’ age group; and 5) healthcare providers involved in PRN medicines management.

To ensure that the data extraction table could gather the required information on the characteristics of selected studies, a pilot test was conducted on a couple of included studies. The review findings were presented narratively, because the presence of heterogeneities in the methods, aims, and results of the studies hindered us to conduct meta-analysis. Therefore, the findings of the included studies were reviewed and based on diversities and similarities in their findings, appropriate categories were developed. The authors discussed to reach agreement on the allocation of the studies’ findings into the relevant categories.

## 3 Results

### 3.1 Search Results and Selection of Studies

The search results on the databases were reported in Table 1. In total, 4,972 articles were retrieved. After removing irrelevant and duplicate titles and carrying out abstract and full-text readings, 31 studies were picked out to be considered for data analysis and research synthesis.

**TABLE 1:** The result of search process.

Search Keyworks	Databases	Total in each database	Selection based on title	Selection based on abstract	Selected based on full text reading	Selection based on quality appraisal and risk of bias assessment
(PRN OR “pro re nata” OR “as needed” OR “as required”) AND (guideline OR “practice guideline” OR “clinical practice guideline” OR “clinical guideline” OR “critical pathway” OR “clinical pathway” OR “critical path” OR “clinical path” OR “patient care planning” OR instruction OR technique OR program*) AND (medication OR drug OR medicines OR “pharmaceutical preparations” OR pharmaceuticals OR “medicines management”)	PubMed (including MEDLINE)	414	33	6	4	4
	Scopus	2,127	49	17	14	14
	Cinahl	1,301	16	0	0	0
	Web of Science	941	17	4	2	2
	*Backtracking references of selected articles*	189	32	23	11	11
	Total	4,972	147	50	31	31

**FIGURE 1:**
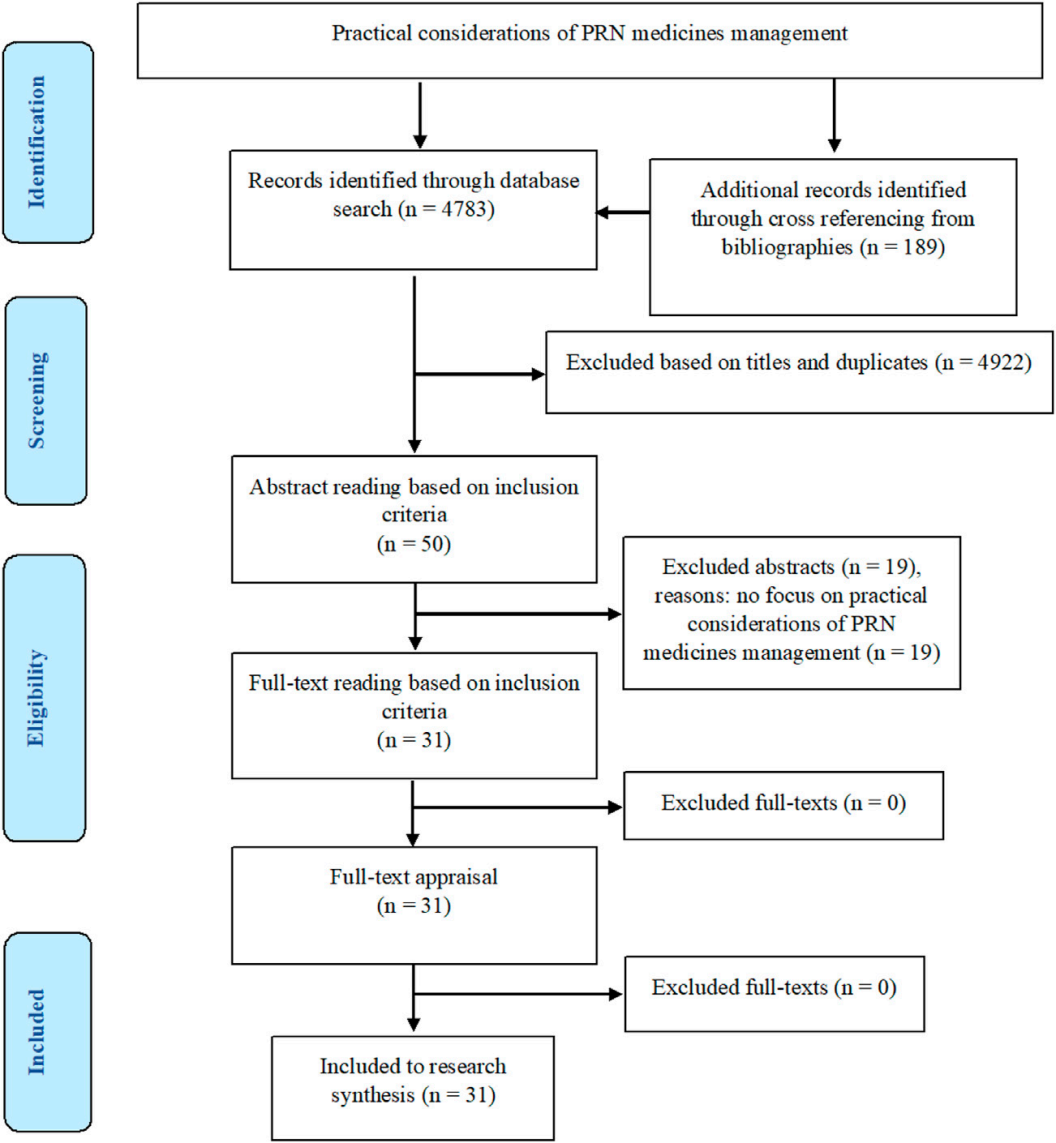
The preferred reporting items for systematic reviews and meta-analyses (PRISMA).

Figure 1 presents the study flow diagram based on the PRISMA.

### 3.2 Quality Assessment and Risk of Bias

The quality appraisal of the selected articles was performed on the full-text of the selected studies (Table 2). Since all studies were judged to have an acceptable level of quality in terms of methodology, theoretical and conceptual framework, no study was ruled out from our review.

**TABLE 2:** General characteristics of the included studies to our data analysis and knowledge synthesis.

Author (year), country	Aim	Methods	Sample and settings	Outcome measurement	Main finding	Conclusion	Quality appraisal
Craven et al. (1987), Canada	To investigate the frequency and indications of the PRN prescription and administration of psychotropic medications in a psychiatric teaching hospital	Chart review	100 patients in general psychiatry wards of a psychiatric teaching hospital	Frequency and indications of PRN prescription and administration	88 patients had PRN prescription (total: 1,041); 75 patients received PRN administration (total: 1,522); diagnosis of personality disorder and age ≥50 years significantly associated with PRN prescription and administration	Hospitals should monitor PRN psychotropic medications use among inpatients and discover reasons for such use; instructions for PRN prescriptions should be obvious and detailed	STROBE Statement/14 from 34
Di Giulio and Crow (1997), United Kingdom	To describe cognitive processes used by nurses and doctors to decide on the administration of PRN analgesics to postoperative cancer patients	Descriptive- comparative	5 nurses and 5 doctors in an oncological digestive surgery department	Cognitive processes used when deciding to administer PRN analgesics to postoperative cancer patients	Wider use of theory and/or experience as the source of information by doctors compare to nurses	Doctors’ main concern was to make the right diagnosis, but the nurses’ main concerns were patients’ reactions and collaboration	STROBE Statement/15 from 34
Edwards et al. (2001), Australia	To investigate the effect of the Peer Intervention Program on nurses’ beliefs, attitudes, subjective norms, self-efficacy, perceived control, and intentions in the management of pain using PRN narcotic analgesia	Quasi-experimental	61 nurses in 21 surgical wards spread across four hospitals	Beliefs, attitudes, subjective norms, perceived control and intention in relation to the management of pain using PRN narcotic analgesia	The peer intervention program changed nurses’ beliefs, self-efficacy, and perceived control in relation to the administration of PRN narcotic analgesia to patients with pain	To improve pain management, a pain management educational program through the utilization of peers can be adopted	CONSORT 2010 checklist/21 from 37 (5 items were N/A)
Geffen et al. (2002), Australia	To examine the knowledge and beliefs of doctors and nurses in inpatient psychiatric units about PRN medications for psychotic disorders	Cross-sectional	80 nurses and 47 doctors in two inpatient psychiatry units	Knowledge and beliefs about PRN medications for psychotic disorders	Nurses selected more indications for PRN antipsychotics than doctors; doctors selected more indications for PRN benzodiazepines	Educational interventions should be devised for both nurses and doctors to achieve the best practice in PRN medication use	STROBE Statement/18 from 34
Baker et al. (2007a), United Kingdom	To explore expert opinion concerning issues and the best practice for the prescription and administration of psychotropic PRN medications within acute inpatient mental health settings	Delphi technique	18 experts (four psychiatrists, 13 nurses and a pharmacist) *via* online discussions	The best practice for the prescription and administration of psychotropic PRN medications within acute inpatient mental health settings	13 clinical practice recommendations were established	Generated items provide useful and practical guidance for prescribers and administrators of PRN psychotropic medications	STROBE Statement/18 from 34
Curtis et al. (2007), Australia	To explore the occurrence of PRN medication administration and the type of alternative therapeutic interventions that are documented as accompanying its administration	Retrospective chart review	64 patients in a mental health facility in an acute admission unit	Occurrence of PRN medication administration, the type of alternative therapeutic interventions that are documented as accompanying PRN administration	47 patients (73.4%) received PRN medications at least once; for nearly three-quarters (73%) of PRN medication administrations, no other therapeutic intervention was documented as occurring prior to administration	Teaching patients and nurses to learn individual techniques to recognize and cope with symptoms than rely on medication as a quick fix	STROBE Statement/17 from 34
Chaichan (2008), Thailand	To evaluate the use of the Positive and Negative Syndrome Scale-Excited Component (PANSS-EC) to evaluate the control of agitation and aggression among inpatients with schizophrenia as a criterion for the administration of PRN medications	Retrospective review of medical records	35 patients prior to the use of PANSS-EC scores/41 patients after its use in two acute inpatient adult psychiatric units	Assessing the effect of adoption of the PANSS-EC as a criterion for the administration of PRN medications for agitation	No statistically significant difference in the mean number of doses of PRN medication administered for agitation before and after adopting the PANSS-EC; lower number of episodes of aggression in the group assessed with the PANSS-EC	The use of criteria based on PANSSEC scores for decision-making for administering psychotropic medications to agitated patients with schizophrenia	STROBE Statement/20 from 34
Gordon et al. (2008), United States	To document nurses’ opinions of the appropriate implementation of PRN opioid analgesic orders for acute pain	Cross-sectional	602 nurses in an academic medical center and a multihospital system with five operating units	Opinions of appropriate analgesic administration practices	Participants mainly chose appropriate responses; attending pain management courses associated with appropriate responses, sedation level, pain intensity rating, respiratory rate, and the patient’s prior response to dosing choose to be considered in opioid administration	Significance of conducting a multidisciplinary examination of range order practices and the need to educate prescribers in how to write appropriate range orders and nurses in how to implement them to provide effective and safe analgesic	STROBE Statement/20 from 34
Stein-Parbury et al. (2008), Australia	To provide a detailed description of circumstances surrounding the use of PRN medications	Retrospective chart review	420 patients in four inpatient units	Prescriptions and administrations of PRN medications	97% were prescribed PRN medications and benzodiazepine was the most frequently prescribed one; 84% received at least one PRN medication; agitation was the most common reason for PRN administration	PRN medication use has endured as standard practice; the combination of second-generation antipsychotics as regular medications and benzodiazepines for PRN medication is consistent with recommended treatment guidelines	STROBE Statement/18 from 34
Kaur et al. (2009), Australia	To examine psychiatric nurses’ responses to patients’ requests for PRN medications and to examine whether these requests were interpreted as “drug-seeking”	Retrospective chart review	38 patients in a secure inpatient hospital	Patients’ history of drug use, the frequency with which they requested PRN medications, how often staff administered PRN medications following requests, and how often patients were labelled “drug seeking”	44.7% of patients were described as ‘drug-seeking’; patients with the history of amphetamine and opiate use were more frequently labelled “drug-seeking”	Need to education to highlight the influence of negative causal attributions on helping behaviours; provision of guidelines to improve the practice of PRN medication administration	STROBE Statement/19 from 34
Usher et al. (2009), Australia	To explore the medical and nursing decision-making process associated with the prescription and administration of PRN psychotropic medications	Qualitative	16 nurses and 3 doctors in three mental health units	Decision-making process associated with the prescription and administration of PRN psychotropic medications	Decision-making processes, factors influencing the administration and prescription of “as needed” medications, individual protocols, improving practice	Need to in-service education for mental health nurses on psychotropic medications and PRN medications; extensive review of PRN medication prescription and administration compared to best practice guidelines	SRQR/17 from 20
Usher et al. (2010), Australia	To explore doctors’ and nurses’ decision making surrounding appropriate PRN psychotropic administration practices within inpatient mental health settings	Qualitative	16 nurses and 3 doctors in three mental health units	Decision-making process associated with the prescription and administration of PRN psychotropic medications	Checking patients’ physical health prior to the administration of PRN medications, caution about administering psychotropic drugs to elderly people, de-escalation prior to a range of further PRN medications	Decisions regarding PRN medication administration are often based upon previous experiences and levels of knowledge. Variable practices associated with when, how much and which drug to administer	SRQR/12 from 20
Mullen and Drinkwater (2011), Australia	To report the rate of PRN medication use in a psychiatric intensive care unit	Retrospective chart review	A psychiatric intensive care unit	Trends in the overall rate of PRN medication administration, time of administration, and type of medication given during the study period	A gradual decline in the total number of given PRN medications, but the typical number of patients per month receiving any PRN did not change	Offering noteworthy insights into the situations that can allow nurses to routinely investigate alternatives to PRN medications and save PRN to a minimum	STROBE Statement/15 from 34
Swart et al. (2011), Canada	To identify patterns for the use of PRN medications given PRN or statim and their efficacy in controlling aggressive behaviors in the mental health services environment	Retrospective chart review	338 youth in a regional children’s MH center	PRN or statim medications were given to control aggressive behaviours	Those youth who received PRNs had a significantly longer period of residential treatment. Those in the Axis II program and had a developmental disability were more likely to receive PRN medications	The Axis II diagnosis of mental retardation in youth influences reasons for the administration of PRN medications, the level of supervision during PRN medication administration, and the total number of times of receiving PRN	STROBE Statement/22 from 34
Carder (2012), United States	To identify how unlicensed staff members decide to administer PRN medications prescribed to the residents of assisted living settings designated for persons with dementia	Qualitative	16 med aides in 3 assisted living	Decision-making regarding the administration of PRN medications	Residents’ request, interpretation of resident-specific behaviours, experience and training, setting-specific practices to guide med aides’ decisions regarding PRN medication administration	Training should identify the implicit knowledge of practicing medication aides; need to understand how other healthcare providers are involved in medication treatment	SRQR/17 from 20
McCarthy et al. (2013), United States	To investigate the patient-centered PRN label instructions, referred to as “Take-Wait-Stop,” versus standard label	Experimental	87 patients in an emergency department	Incorrect dosing	Use of the Take-Wait-Stop label caused a reduction in going beyond the maximum daily dose	Use of the Take-Wait-Stop method significantly reduces maximum daily dose	CONSORT 2010 checklist/16 from 37 (6 items were N/A)
Akram et al. (2014), Scotland	To determine the frequency and nature of PRN practice	Retrospective chart review	75 patients in 10 psychiatric intensive care units	Frequency and nature of PRN practice	The most frequently administered PRN medication were lorazepam, haloperidol, and zuclopenthixol; the mean number of PRN administrations per patient per day was 0.4	Inadequate monitoring and documentation of PRN medications; possible insufficient understanding of prescribers regarding differences in bioavailability between oral and injectable forms of medications	STROBE Statement/16 from 34
Al-Sughayir (2014), Saudi Arabia	To investigate whether the mental health accreditation program drives improvements in the clinical practice of giving PRN antipsychotic medications for psychiatric inpatients	A record-based pre-post assessment	177 patients during the pre-accreditation period/182 patients during the post-accreditation period in a psychiatric inpatient adult unit	Number of PRN antipsychotic medications administered and indications for use	12.10 ± 7.0 and 7.47 ± 3.2 of PRN antipsychotics were administered per patient pre- and post-accreditation, respectively	Implementation of clinical practice guidelines during the mental health accreditation program significantly reduces the frequency of PRN antipsychotic medications and can enhance patient safety	STROBE Statement/14 from 34
Russell et al. (2014), Australia	To document PRN prescribing practices and to identify patterns with respect to clinical characteristics and medications prescribed	Prospective consecutive case note review	203 individuals in two hospices and palliative care services	PRN prescribing practices and associated factors	Mean number of PRN medications prescribed was 3.0. Higher rates of PRN medications in the last week of life and during the terminal phase of disease was observed	The trends of increasing numbers of PRN prescriptions and worsening the clinical status show the flexibility in prescribing PRN medications and to respond rapidly to changing clinical symptoms or circumstances	STROBE Statement/20 from 34
Dörks et al. (2016), Germany	To examine characteristics of PRN drug use and potential predictors in nursing homes	A cross-sectional study	852 residents in 21 nursing homes	Characteristics and potential predictors of PRN medication use	74.9% of residents received at least one PRN medication; more length of stay and polypharmacy, with five or more long-term medications were associated with a higher number of PRN prescriptions	Physicians should regularly review the need for any PRN medication in the medication plan. The high prevalence of PRN medications and its relationship with the length of stay underscore the importance an accurate documentation	STROBE Statement/26 from 34
Al-Sughayir, (2017), Saudi Arabia	To investigate whether hospital accreditation drives improvements for administered PRN benzodiazepines in psychiatric inpatients	Record-based pre-post assessment	177 patients during the pre-accreditation period/182 patients during the post-accreditation period in a psychiatric inpatient adult unit	Number of administrations of PRN benzodiazepines	Average number of PRN benzodiazepines’ administrations per patient post-accreditation was 4.83 ± 2.1 compared to 6.19 ± 3.4 pre-accreditation	Accreditation may have a positive influence on the process of administering PRN benzodiazepines’ medications in psychiatric inpatients	STROBE Statement/17 from 34
Barr et al. (2018), Australia	To identify mental health nurses’ attitudes towards the use of PRN medications with mental health consumers in a forensic and non-forensic acute mental health setting in Australia	Survey	70 nurses in three acute mental health units	Nurses’ attitudes towards the use of PRN medications with mental health consumers	Practice differences between forensic and other acute mental health settings were related to the use of PRN medications to manage symptoms from nicotine, alcohol and other drug withdrawals, use of comfort rooms, and conducting comprehensive assessments of consumers’ psychiatric symptoms	Need for services for regular monitoring and reviewing medication prescribing and administration practices at the service level to reduce reliance on PRN medication administration	STROBE Statement/19 from 34
Martin et al. (2018a), Canada	To describe and compare the documentation of PRN medications for anxiety at two psychiatric hospitals, one that used paper charts and another that used electronic health records; to examine congruence between nursing documentation and their verbal reports	Mixed-methods	400 administrations of PRN medications for anxiety in two psychiatric hospitals	Documentation of PRN medications for anxiety; congruency between nursing documentation and their verbal reports	Nurses using electronic health records documented more information in comparison to those using paper charts. There were some diversities between written and verbal reports	Calls for improving the quality of nursing documentation; supporting the shift to the use of electronic health records	GRAMMS/4 from 6
Stasinopoulos et al. (2018), Australia	To determine the frequency of, and factors associated with PRN medication administration in residential aged-care services	Secondary analysis of cross-sectional data	383 residents in 6 residential aged-care services	Frequency and factors associated with PRN medication administrations	94% residents charted ≥1 PRN medication and 99 (28%) were administered PRN medications at least once; residents with greater dependence with the activities of daily living and a greater number of regular medications were more likely to be administered PRN medication	The portion of PRNs to medication burden in residential aged care services may be lower than previously thought	STROBE Statement/20 from 34
Griffiths et al. (2019), United Kingdom	To describe the prescription and administration rates of PRN medications for people with dementia in United Kingdom care homes	Cross-sectional study	728 participants with dementia or memory problems in 50 care homes	Prescription and administration of PRN medications for the treatment of behaviours associated with neuropsychiatric symptoms and pain	The total number of PRN medication prescriptions was 317. The most commonly prescribed PRN medications (35.3%) were analgesics	Low levels of medication prescriptions and even lower levels of administrations are observed for the management of neuropsychiatric symptoms	STROBE Statement/23 from 34
Jimu and Doyle (2019), Ireland	To explore the process of PRN medication administration by mental health nurses	Qualitative	19 nurses in an acute inpatient service	Process of PRN medication administration	Undertaking an assessment of the patient before administering PRN medications; need for service improvements in terms of the use of alternative strategies than PRN use	There is a potential for improvement in relation to how PRN medications is prescribed and administered	SRQR/17 from 20
McCarthy et al. (2019), United States	To assess the implementation of a patient-centered PRN label entitled Take-Wait-Stop (TWS) with three deconstructed steps replacing traditional wording	Experimental	211 patients in an emergency department	Prescriptions labels	12% one step wording; 26% two-step wording; 44% three-deconstructed steps	Higher implementation reliability for new instructions such as Take-Wait-Stop (TWS) requires additional supports	CONSORT 2010 checklist/18 from 37 (6 items were NA)
Nilsen et al. (2020), Norway	To describe healthcare personnel perceptions of factors affecting PRN medicines management in sheltered housing for older adults	Qualitative	22 healthcare personnel in sheltered housing from four municipalities representing urban, suburban and rural districts	Factors affecting PRN medicines management	Four main factors including the medication, the resident, the healthcare personnel, and the organisation affecting PRN medicines management	Safe PRN medicines management requires inter-professional collaboration and professional practice with appropriate medical competence and knowledge, practical experience and skills, and communication and documentation competency	SRQR/20 from 20
Procaccini et al. (2020), United States	To increase compliance of PRN sedative and analgesic orders with the use of failure mode and effects analysis and human factors risk assessment methodologies in a pediatric intensive care unit	Quality improvement	A pediatric intensive care unit	Proportions of compliant PRN analgesic and sedative orders based on the Joint Commission Medication Management standards	After staff education, weekly average PRN orders compliance increased from 62.0 to 77.7%; after order set implementation, weekly average compliance further increased to 93.2%	Interdisciplinary collaboration and a combined failure mode and effects analysis and human factors risk assessment are effective strategies for identifying the failure modes of PRN medication orders	CONSORT 2010 checklist/12 from 37 (6 items were NA)
Walsh et al. (2020), Canada	To understand how acute care nurses make decisions about administering PRN psychotropic medications to hospitalised people with dementia	Qualitative	8 nurses in three medical units	Decision making about administering PRN psychotropic medications to hospitalised people with dementia	Legitimising control (medicating undesirable behaviours to promote the nurses’ perceptions of safety), making the patient fit (maintaining routine and order), and future telling (pre-emptively medicating to prevent undesirable behaviours from escalating) were developed	Need for better understanding of how to improve nursing practice in relation to PRN medication administration to hospitalised people with dementia	SRQR/17 from 20
Sharma et al. (2021), Australia	To determine the prevalence and factors associated with PRN medication administration in residential aged-care facilities and examine changes over 12-months	Secondary analysis	242 residents in 8 residential aged care facilities	Prevalence and factors associated with PRN medication administration	87.2% residents were prescribed ≥1 PRN medication; PRN administration was less likely among residents with more severe dementia symptoms and greater dependence with activities of daily living	Contribution of PRN medications to entire medication use in residential aged-care facilities is small and PRN is relatively static over 12-months	STROBE Statement/22 from 34

The results of risk of bias evaluation for two randomized controlled studies (McCarthy et al., 2013; McCarthy et al., 2019) were presented in Supplementary Figure S1. In terms of bias in random sequence generation, allocation concealment, blinding of participants and personnel, blinding of outcome assessment, and selective outcome reporting, the studies presented insufficient data leading to the judgement of unclear risk of bias. In addition, they were judged to have a low risk of bias in terms of bias in incomplete outcome data.

The risk of bias assessment in quasi-experimental (Edwards et al., 2001) and qualitative studies (Procaccini et al., 2020) were described in Supplementary Figure S2. In terms of bias due to confounding and selection of participants into the study, one study was judged to have a low risk of bias and another one had a serious risk of bias. In terms of bias in the classification of interventions and bias due to missing data, one study was judged to have a low risk of bias and another had a critical risk of bias. In addition, both studies had low risk of bias in the view of bias due to deviations from intended interventions and failure to provide information in terms of bias in the measurement of the outcome and bias in the selection of the reported result.

Furthermore, the results of risk of bias for 20 observational studies were presented in Supplementary Figure S3. The selected studies mostly had a low risk of bias in terms of the assessment of exposure (100%), development of the outcome of interest (95%), selection of cases (85%), and controls (85%). In terms of the control of prognostic variable, 40% of the studies had low risk of bias, 20% probably low risk of bias, 15% high risk of bias, and 25% probably high risk of bias.

### 3.3 Characteristics of Selected Studies

A summary of selected studies (*n* = 31) has been presented in Table 2. All studies published in English from 1987 until 2021. Twelve studies were from Australia (Edwards et al., 2001; Geffen et al., 2002; Curtis et al., 2007; Stein-Parbury et al., 2008; Kaur et al., 2009; Usher et al., 2009; Usher et al., 2010; Mullen and Drinkwater, 2011; Russell et al., 2014; Barr et al., 2018; Stasinopoulos et al., 2018; Sharma et al., 2021), five from the United States (Gordon et al., 2008; Carder, 2012; McCarthy et al., 2013; McCarthy et al., 2019; Procaccini et al., 2020), four from Canada (Craven et al., 1987; Swart et al., 2011; Martin et al., 2018a; Walsh et al., 2020), three from the United Kingdom (Di Giulio and Crow, 1997; Baker et al., 2007a; Griffiths et al., 2019), two from Saudi Arabia (Al-Sughayir, 2014; Al-Sughayir, 2017), one from Germany (Dörks et al., 2016), one from Ireland (Jimu and Doyle, 2019), one from Norway (Nilsen et al., 2020), one from Scotland (Akram et al., 2014), and one from Thailand (Chaichan, 2008).

Regarding their methods, 11 studies used the chart review method (Craven et al., 1987; Curtis et al., 2007; Chaichan, 2008; Stein-Parbury et al., 2008; Kaur et al., 2009; Mullen and Drinkwater, 2011; Swart et al., 2011; Akram et al., 2014; Al-Sughayir, 2014; Russell et al., 2014; Al-Sughayir, 2017), six were cross-sectional (Di Giulio and Crow, 1997; Geffen et al., 2002; Gordon et al., 2008; Dörks et al., 2016; Barr et al., 2018; Griffiths et al., 2019), six were qualitative (Usher et al., 2009; Usher et al., 2010; Carder, 2012; Jimu and Doyle, 2019; Nilsen et al., 2020; Walsh et al., 2020), three were interventional (Edwards et al., 2001; McCarthy et al., 2013; McCarthy et al., 2019), two were secondary analysis (Stasinopoulos et al., 2018; Sharma et al., 2021), one was Delphi technique (Baker et al., 2007a), one was mixed-methods (Martin et al., 2018a), and one was qualitative improvement (Procaccini et al., 2020).

### 3.4 Practical Considerations of *Pro re nata* Medicines Management

Characteristics of PRN medicines management including name and dose of PRN medications, patient’s age group, healthcare providers who were involved in PRN medicines management, and related practical considerations for each of the included studies were presented in Table 3.

**TABLE 3:** PRN medicines management and related practical considerations based on the findings of each included study.

Author, year, country	Name and dose of PRN medications	Patient’s age group	Healthcare providers involved in PRN medicines management	Practical considerations
Craven et al. (1987), Canada	Total PRN prescriptions: 1,041; most prescriptions were neuroleptics (32%), antiparkinsonians (31%) and sedative-hypnotics (30%); total PRN administration: 1,522; most administrations were neuroleptics (32%), antiparkinsonians (17%), and sedative-hypnotics (45%)	Men: 34 years (range = 17 to 69 years)	Nurses and physicians	The use of specifically designed sheet for PRN medicines management for medication name, dose, route of administration, and a space for the physician’s instructions; use of stop-order policy after 7 days, reassessment of prescription needs by the physician; documenting the reason for PRN medication administration; specifying the indication for PRN prescriptions; stating the time interval between the doses of PRN medications and maximum dosage limit per 24 h during medication prescriptions; deprescribing PRN medications when they are no longer needed
Di Giulio and Crow (1997), United Kingdom	Analgesics	N/A	Nurses and physicians	Making decisions on PRN based on collected data; relying on theoretical and practical knowledge for PRN medication prescription and administration; consideration of patient’s symptoms, behaviours, and preferences for PRN use; consideration of laboratory test results; collecting data on vital signs; having a closer look at psychological symptoms and a broader perspective rather than problem-specific for medication use; being worried about the administration of wrong medications that can hamper diagnosis; interference of medications in patient’s collaboration with the treatment plan
Edwards et al. (2001), Australia	Narcotics	N/A	Nurses	Having a positive attitude toward the administration of PRN medications; having a good intention for PRN medication administration; positive attitude by the patient, family members and healthcare providers toward PRN medications use; ability to administer PRN medications
Geffen et al. (2002), Australia	Antipsychotics, benzodiazepines, anticholinergics	N/A	Physicians and nurses	Use of both subjective (internal state) and objective (behaviour) assessment methods to make decisions on medication use; consideration of alternative interventions instead of PRN medications
Baker et al. (2007a), United Kingdom	Psychotropic medications	N/A	Physicians, nurses and pharmacists	Clear purpose for PRN medications; being aware of the potential side effects of PRN medications; ensuring the match between the indication for PRN prescription and administration; consideration of side effects and additional medication interactions/allergic reactions; finding allergies prior to administration; having the clear goal underpinning the use of PRN medications; clear description of indications for PRN; joint decision making about the prescription wherever possible –including translating/agreeing the rational/indication for the prescription into the language of/with the service user; time-limited prescription of PRN medications, with regular reviews; gaining knowledge of any advance directive related to PRN medications; clear documentation of circumstances leading to the administration of PRN medications and its beneficial or detrimental impact on behaviour; regular and systematic evaluation of the use and effects of PRN medications for individual patients; communicating the rational to the service user as well as information about any perceived risks, answering questions, and seeking consent
Curtis et al. (2007), Australia	309 psychotropic medications were administered on 268 occasions including	<19: 8	Nurses	PRN administrations based on rational and reason; documentation of PRN medication effects; description of the method used for the evaluation of PRN medication effects; documentation of any additional pre- or post-intervention when PRN medications are used
	1. Benzodiazepines (*n* = 188, 60.8%)	20–29: 12		
	2. Atypical antipsychotic (*n* = 31, 10%)	30–39: 17		
	3. Typical antipsychotic (*n* = 87, 28.1%)	40–49: 18		
	4. Other (*n* = 2, 0.6%)	50+: 9		
Chaichan (2008), Thailand	Psychotropic medications for agitation in patients with schizophrenia; prescriptions: all study participants were prescribed at least one PRN medication; the most frequently prescribed medication was haloperidol in the control group and in the Positive and Negative Syndrome Scale-Excited Component (PANSS-EC) group	Mean (SD): control group: 32.49 (8.67) years	Nurses and physicians	Inclusion of the medication name, dose, route of administration, reason for use, and shortest time allowed before and the dose can be repeated in the physician order; use of assessment tools during admission to determine the need for PRN medications, avoiding PRN administration when the minimum time specified between doses of the medication is violated
	Administration: in the control group, 23 patients (65.7%) received 54 doses of PRN psychotropic medications, while 23 patients (56.1%) in the PANSS-EC group received 56 doses	PANSS-EC group: 35.54 (9.33) y		
Gordon et al. (2008), United States	Opioids	N/A	Nurses	Consideration of the sedation level, pain intensity, respiratory rate, prior response for the selection of opioids; paying attention to the interval and dose of the re-administration of a similar PRN medication
Stein-Parbury et al. (2008), Australia	97% of the patients (408/420) were prescribed PRN medications; total prescription: 139. The most frequently prescribed medications	Mean: 38.63 years	Nurses and physicians	Administration of more than one PRN medications without the description of its clear indication; documentation of the indication of the administration of PRN medications; documentation of the outcome of PRN medication administration
	1. Benzodiazepines (52.2%)			
	2. First-generation antipsychotic (FGAs): 16.6%			
	administrations			
	for 420 admissions, 3,868 episodes of PRN medications; types of administrated			
	1. Benzodiazepines: 70.7%			
	2. FGAs: 18.1%			
	3. Benztropine: 4.3%			
Kaur et al. (2009), Australia	N/A	N/A	Nurses	Assessing the drug dependency and abuse
Usher et al. (2009), Australia	N/A	N/A	Nurses and physicians	Consideration of the patient’s behaviour, concerns and requests; having concerns about the prescription of the new atypical medications as PRN; PRN medication use only after trying alternatives; not interpreting the patient’s request as the drug-seeking behaviour; prescription and administration based on thorough assessment of patients and getting knowledge of his/her background; concerns about ineffectiveness of medications, and related side effects; being looked a like unwell to receive PRN medications; patient’s willingness and previous effectiveness to choose alternative methods; severity of the patient’s health condition and symptoms as the factor affecting medication use; staffing pattern and shortages and inexperienced staff to affect the medication use; personal perspective and philosophy by nurses for PRN medication use; presence of the individual medication protocol to decide on PRN medication administration; need to clear and up-to-date prescription information; being ensured of patient safety in the caring environment; clear writing of medication orders by the doctor
Usher et al. (2010), Australia	Psychotropics	N/A	Nurses and physicians	Regular patient’s checking in terms of physical health before and after medication use; de-escalation using restraints and seclusion before PRN medication use
Mullen and Drinkwater (2011), Australia	50–60% of patients in the psychiatric intensive care unit received at least one PRN medication during their stay; the most frequently administered PRN medication during all four periods was diazepam	N/A	Nurses	Timing of PRN medication use
Swart et al. (2011), Canada	50.3% of patients received one or more PRNs; three most medications were chlorpromazine, lorazepam, and olanzapine	Mean SD: 12.3 years (2.68)	Nurses and physicians	Assessing techniques for reducing PRN medication use including counselling, prompt to calm, redirection, planned ignoring, offering alternative choices, and reminder of consequences; assessing the reason for PRN medication administration such as gesture of treat
Carder (2012), United States	N/A	N/A	Med aides	Expression of symptoms and request for medications by the patient; provision of instructions with enough detail for the appropriate use of PRN medications such as the dosage guideline; provision of training to healthcare providers in relation to PRN medications; giving information in relation to patients’ medication during shift handoff interpreting the patient’s non-verbal behavioural clues; regulations for PRN medications use in terms of reasons for use, schedule and route, circumstances for use, maximum dose, when to call the resident’s physician, and when to discontinue; appropriate storage of medications to facilitate access to medications
McCarthy et al. (2013), United States	Pain medication containing acetaminophen	Mean (SD): 39.8 (12.9)	Pharmacists	Use of the Take-Wait-Stop label design consisting of explicit, deconstructed instructions and simplified text (numeric characters instead of words, e.g., “1 tab” instead of “one tab”, and “carriage returns” to place each part of the instructions on separate lines; use of word “stop” instead of “do not exceed” to convey the maximum daily dosage to patients in plain language; deconstructing instructions so that each action or intended behavior was separate and would potentially allow patients to be more cognizant of each step to be taken
Akram et al. (2014), Scotland	65% of patients were administered psychotropic PRNs (total 396 doses); number of most frequently administrated psychotropic PRNs	Male patients: 37 years; female patients: 40 years	Nurses	Patient’s request for medications or nurses’ decision making on PRN; assessing the peak time of medication administration in the day; administration route of medications; assessing the reason of PRN medication use as rapid tranquilisation; simultaneous use of PRN medications and restrains; documentation of post medication administration monitoring
	1. Oral forms of lorazepam (*n* = 198)			
	2. Oral form of haloperidol (*n* = 66)			
	3. Oral form of zuclopenthixol (*n* = 22)			
	4. Injection form of lorazepam and quetiapine (both *n* = 14)			
Al-Sughayir (2014), Saudi Arabia	Antipsychotics	<25: 109 (30.3%)	Nurses and physicians	Reconciliation of medications soon after patient admission and their documentations; use of regular medications for individual patients as PRN; avoiding polypharmacy; consideration of alternative methods such as counselling when handling the patient’s difficult behaviour before resorting to PRN medications; completing PRN regimen order among the treating psychiatrist as soon as possible; use of oral PRN medications when the patient accepts them and when the required response is achieved rather than injections; documenting administered PRN medications and the patient’s response to them; monitoring vital signs for side effects such as extrapyramidal side effects after administering PRN medications; informing the treating psychiatrist and asking for a medical evaluation in case of any concern
		25–50: 208 (57%)		
		>50: 42 (11.7%)		
Russell et al. (2014), Australia	606 total PRN prescriptions including	Mean (SD): 72.9 years (12.6 years)	Physicians and nurses	Assessment of polypharmacy and over-prescription of medications; considering inappropriate PRN medications prescription; prescribing and administering PRN medications according to the patients’ condition
	1. Opioid: 178 (29.4%)			
	2. Antiemetic: 112 (18.5%)			
	3. Benzodiazepine: 99 (16.3%)			
	4. Laxative: 82 (13.5%)			
	5. Acetaminophen: 56 (9.2%)			
	6. Other: 79 (13%)			
Dörks et al. (2016), Germany	Total 2117 PRN prescriptions; most commonly used PRN drugs, *n* (%)	Mean (SD): 83.5 years (10.5 years)	Physicians and nurses	Monitoring the number of medications in patients with a long duration of hospitalisation
	1. Acetaminophen: 299 (14.1%)			
	2. Metamizole: 272 (12.8%)			
	3. Ibuprofen: 124 (5.9%)			
	4. Macrogol: 110 (5.2%)			
	5. Loperamide: 103 (4.9%)			
	6. Lactulose: 101 (4.8%)			
	7. Melperone: 84 (4.0%)			
	8. Metoclopramide: 74 (3.5%)			
	9. Lorazepam: 69 (3.3%)			
	10. Bisacodyl: 60 (2.8%)			
Al-Sughayir, (2017), Saudi Arabia	Benzodiazepine	<25: 109 (30.3%)	Nurses and physicians	Reconciliation and documentation of current medications after admission; use of regular medications for individual patients as PRN; avoiding polypharmacy; consideration of alternative methods such as counselling when handling the patient’s difficult behaviour before resorting to PRN medications; completing the PRN regimen among the treating psychiatrist as soon as possible; use of oral PRN medications when the patient accepts them and when the required response is achieved rather than injections; documentation of administered PRN medications and the patient’s response to it; monitoring vital signs for side effects after PRN medication administration; informing the treating psychiatrist and asking for a medical evaluation in case of any concern
		25–50: 208 (57%)		
		>50: 42 (11.7%)		
Barr et al. (2018), Australia	Psychotropics	N/A	Nurses	Consideration of underlying diagnosis in PRN prescription and administration; attention to the patient’s request for PRN medications; use of PRN medications for reducing agitation in patients who are unable to follow their previous behaviours such as smoke cigarettes, drink alcohol or access illicit drugs; accurate assessment *via* appropriate tools to determine the need for PRN medications and reduce PRN medication use; consideration of alternative methods such as music and relaxation to reduce PRN use; regular medication prescription instead of PRN orders; multidisciplinary team collaboration for the management of behaviour and reduction of medication use; taking more responsibility in the prescription and administration of PRN medications and being aware of issues resulting from high dose and poly-pharmacy; combination of PRN medications with other methods to improve its effectiveness; use of PRN medications to reduce the use of seclusion and restrictive measures damaging therapeutic relationships; use of alternative methods such as behaviour therapy and relaxation instead of PRN to improve self-care
Martin et al. (2018a), Canada	400 administrations of PRNs for anxiety; 80% of the prescriptions were lorazepam	—	Nurses	Identification and documentation of symptoms related to the need for PRN medication use; documentation of PRN medications when it is administered; documentation of the reason for PRN medication administration; documentation of the effect and side effect of PRN medications; trying non-pharmacological interventions prior to administering PRN medications
Stasinopoulos et al. (2018), Australia	94% of residents were charted at least one PRN medication (median: 4); the most prevalent charted PRN medications, number of residents who had charted PRN (%) were	Median (interquartile range (IQR): 88 (84–92)	Nurses and physicians	Assessment of over-medication and polypharmacy; assessment of over-prescription of PRN medications in patients with more dependency levels
	1. Paracetamol: 178 (46.5)			
	2. Docusate sodium ± senna: 143 (37.3)			
	3. Bisacodyl: 82 (21.4)			
	4. Oxycodone: 72 (18.8)			
	5. Metoclopramide: 72 (18.8)			
	6. Glyceryl trinitrate: 69 (18.0)			
	7. Macrogol: 62 (16.2)			
	8. Betamethasone: 56 (14.6)			
	9. Temazepam: 54 (14.1)			
	10. Oxazepam: 50 (13.1)			
	11. Salbutamol (inhaled): 49 (12.8)			
Griffiths et al. (2019), United Kingdom	317 PRN prescriptions; 180 PRN medications were administrated	Mean (SD): 85.6 years (7.64 years)	Physicians and nurses	Evaluation of the effects of PRN medications on the symptoms of the underlying health conditions; consideration of polypharmacy with PRN medications administration; association between the severity of the symptoms experienced and the amount of prescribed PRN medications
	1. Antipsychotic, 10 prescribed, 2 administrated			
	2. Benzodiazepine, 39 prescribed, 19 administrated			
	3. Non-benzodiazepine hypnotic, 6 prescribed, 5 administrated			
	4. Antidepressant, 3 prescribed, 3 administrated			
	5. Analgesic, 259 prescribed, 151 administrated			
Jimu and Doyle (2019), Ireland	N/A	N/A	Nurses	Assessing the patient in terms of physical and psychological symptoms; undertaking a risk assessment with regard to the patients and others; preparing the patient with regard to when PRN medications should be administered; discussing changes in PRN medication use between the physician and nurse; consideration of over-medication and poly-pharmacy; consideration of alternative treatment methods; need for senior nurses to get involved in the PRN medication process and discuss administration
McCarthy et al. (2019), United States	Hydrocodone-acetaminophen	Mean (SD): 44.3 years (14.3 years)	Pharmacists	Developing the Take-Wait-Stop label, following the patient-centered prescription label design; deconstructing prescription wording regarding the core components of PRN instructions to explicitly convey the dose, interval between doses, and maximum daily dose; PRN instruction emphasis on deconstructing actions and behavioural steps that support understanding and recall; employing numeric characters instead of words, e.g., “1 tab” instead of “one tab,” and “carriage returns” place each section of the instructions on different lines; use of simplified text and plain language, “Stop” to replace the typical wording “do not exceed,” to convey maximum daily dosing among patients with limited literacy
Nilsen et al. (2020), Norway	N/A	N/A	Nurses, healthcare workers, apprentices in health and social work, social educators	Judgement of the patients’ symptoms for PRN medication use; creating a consensus on PRN medication use through interprofessional medication review; patients’ participation in decision making on PRN medications; patients’ knowledge of list of medications; communication and cognitive abilities of patients to assess the necessity of PRN medication use; reaching agreements by the healthcare providers and families on PRN; healthcare staff’s knowledge of medicines management; seeking for complementary competency through asking for the second opinion; significance of practical knowledge; skills for the assessment of the effects of PRN medications; appropriate staffing pattern in the ward; sharing verbal and written information; appropriate storage of medications to facilitate access; culture of medication use as the use of non-pharmacological methods prior to medication use
Procaccini et al. (2020), United States	Sedative and analgesic medications	N/A	Physicians, nurses, pharmacists	Consideration of clinical indications for the use or discontinuation of PRN medications; sequencing PRN medications for the same healthcare problem; communication with prescriber in case of unsuccessful outcome of PRN use; education of healthcare staff to comply with PRN medication standards; use of decision support tools
Walsh et al. (2020), Canada	Psychotropics	N/A	Nurses	Perceived harm and the probability of risk of patients by healthcare providers as the indicator of PRN medication use; patient’s preference and compliance with PRN medication use; use of fast acting medications to prevent patient’s self-harm; close monitoring of the patient’s behaviours and symptoms to find indication for medication use; controlling undesirable behaviours to legitimate PRN medication use; use of non-pharmacologic strategies such as restrain before medication use; use of PRN medications based on the hospital’s protocol to prevent the use of restraints; time-consuming identity of nonpharmacologic interventions such as distraction and redirection; more PRN use due to higher workloads and staff shortages; use of PRN medications to manage sleep disturbances and help adjust with the work unit; PRN medication administration to the best interest of the patients; collective decision making on PRN medications based on the nurse’s perspectives and the patient’s behaviour and symptoms; use of PRN medications based on predicting the patient’s pattern of behaviours and knowing the patient; consideration of the disease’s general pattern and the underlying cause of behaviours for PRN medication use; misinterpretation of the patient’s behaviours due to communication issues and PRN medication use
Sharma et al. (2021), Australia	1090 PRN prescribed; the most prevalent PRN medications prescribed were paracetamol (54.1% of residents), docusate and sennosides (40.9%) and metoclopramide (26.8%)	Median (Interquartile range): 87.0 (81.0–92.0)	Physicians and nurses	Assessment of daily dose recommendation of medications; assessment of the PRN medication administration in patients with severe cognitive issues

During the narrative research synthesis, eight categories in relation to the practical considerations of PRN medicines management were identified: “PRN indications and precautionary measures,” “requirements of PRN prescription,” “interventions for PRN administration,” “monitoring and follow up interventions,” “deprescription strategies,” “healthcare professionals’ role,” “participation of patients and families,” and “multidisciplinary collaboration”. The initial list of items corresponding to each category has been presented in Supplementary File S4.

#### 3.4.1 *Pro re nata* Indications and Precautionary Measures

Prescription of PRN medications should be based on the thorough assessment of patients and collection of data about their medical history (Usher et al., 2009). For the prescription of PRN medications, appropriate indications and purpose of medication use should be specified and fully described (Craven et al., 1987; Baker et al., 2007a). Craven et al. (1987) reported that physicians had not specified any indication for 47% of PRN prescriptions for psychiatric inpatients.

When healthcare providers prescribe a new atypic medication as PRN, they should have more concerns and give more attention to its efficacy and side effects (Usher et al., 2009). Healthcare providers require to find clinical indicators for the continuation or discontinuation of PRN medications in the patient’s treatment plan (Procaccini et al., 2020) and consider underlying health condition and diagnosis for PRN medication prescription (Barr et al., 2018).

The use of regularly prescribed medications in a suitable dose at the time of hospitalization instead of PRN orders has been shown to help with the reduction of the use of PRN medications (Barr et al., 2018). Effectiveness of this method in psychiatric wards has been shown (Al-Sughayir, 2014; Al-Sughayir, 2017).

#### 3.4.2 Requirements of *Pro re nata* Prescription

Medication reconciliation and documentation of current medications should be performed soon after admission to the hospital (Al-Sughayir, 2014; Al-Sughayir, 2017). A specifically designed sheet containing headings for medication name, dose, route of administration, and an empty space for the physician’s instructions should be devised for the prescriptions of PRN medications (Craven et al., 1987). Also, details of the reason for PRN medication use (Chaichan, 2008), the time interval between the doses of medications (Craven et al., 1987; Chaichan, 2008), maximum dosage limit per 24 h (Craven et al., 1987), and sequencing PRN medications for the same healthcare problem, if applicable, (Procaccini et al., 2020), should be specified.

Besides the patient’s health condition, his/her preference should guide healthcare providers for the prescription of appropriate PRN medications. When the patient is at risk of self-harm, the use of fast-acting PRN medications is suggested (Walsh et al., 2020). In addition, the use of PRN medications to reduce agitation in patients who are unable to follow their previous habits including smoke cigarettes, drink alcohol, and access to illicit drugs have been recommended (Barr et al., 2018).

Controlling the undesirable behaviors of patients is a legitimate reason for the prescription and use of PRN medications when non-pharmacological strategies do not work properly (Walsh et al., 2020). Oral PRN medications have been recommended rather than injections when the patient accepts that the required response can be achieved via this method (Al-Sughayir, 2014; Al-Sughayir, 2017).

#### 3.4.3 Interventions for *Pro re nata* Administration

Having clear goals and ration underpinning the use of PRN medications is required (Baker et al., 2007a; Curtis et al., 2007). The study by Curtis et al. (2007) reported that the rationale for the administration of 42% of PRN psychotropic medications within acute mental health settings was not stated. The PRN medication use should be supported with logic and reasons (Akram et al., 2014). It helps healthcare providers ensure the match between the indication of prescription and administration of PRN medications (Baker et al., 2007a). They require to communicate this ration to the patient and families involved in patient care along with the provision of information about any perceived risks. Answers to their questions should be given and their consent before medication administration should be sought (Baker et al., 2007a).

Timing of PRN medication use should be considered (Mullen and Drinkwater, 2011) and PRN administration should be avoided when the specified minimum time between the doses of the medication would be violated (Chaichan, 2008). Adequate attention should be paid to the interval and dose of re-administration of a similar PRN medication (Gordon et al., 2008).

The route of PRN medication administration and its dose are the important aspects of medicines management. Plasma levels of medications from oral ingestion are notably lower than those of an intramuscular or intravascular injection. Therefore, side effects are more likely to happen when the comparative doses of medications are administrated *via* injection instead of oral use (Akram et al., 2014).

Being prepared and having PRN medication orders when a patient is involuntarily admitted and is at risk of harm to themselves or others help healthcare providers to administer medications to control a potentially violent incident (Jimu and Doyle, 2019).

Making a decision on PRN medication administration should be based on collected data and the assessment of patients and their healthcare background (Di Giulio and Crow, 1997; Usher et al., 2009). Accordingly, healthcare providers should monitor patients’ physical and psychological symptoms (Usher et al., 2010; Swart et al., 2011; Jimu and Doyle, 2019; Walsh et al., 2020), check their laboratory test results, evaluate their vital signs (Di Giulio and Crow, 1997), assess their allergies (Baker et al., 2007a), consider their behaviors, concerns and requests (Di Giulio and Crow, 1997; Usher et al., 2009; Barr et al., 2018; Walsh et al., 2020). In this respect, the use of both subjective assessment such as interviewing and objective assessment such as observation help with making decisions on PRN medication use (Geffen et al., 2002). Healthcare providers can make a decision on the administration of PRN medications through the interpretation of the patient’s actions and non-verbal clues (Carder, 2012). Specifically, prior to the administration of opioid medications as PRN, the sedation level, pain intensity, respiratory rate, and prior response to medications should be assessed (Gordon et al., 2008). Severity of the patient’s health condition and related symptoms indicate the need for medications (Usher et al., 2009). Therefore, a collective decision-making on PRN medications can be made based on the healthcare provider’s perspectives and the patient’s behaviors and symptoms (Walsh et al., 2020). Perceived harm and the probability of risk should be detected by healthcare providers as an important indicator of PRN medication administration (Walsh et al., 2020).

There is a need to have a closer look at psychological symptoms and having a broader perspective rather than problem-oriented one for the appropriate use of PRN medications (Di Giulio and Crow, 1997). It helps predict the patient’s pattern of behaviors by knowing the patient and empower him/her to safely use PRN medications for the prevention of dangerous behaviors and related harm (Walsh et al., 2020). Healthcare providers’ judgment of the patient’s symptoms is decisive for PRN medication administration. Some symptoms such as heavy breathing or constipation clearly have an obvious cause, which facilitate decision-making regarding PRN medication use. On the other hand, a single night insomnia is not judged to be an indication for the use of hypnotics as PRN (Nilsen et al., 2020).

Communication challenges between the patient and healthcare providers contribute to the increased use of PRN medications. It hinders the assessment of patients’ underlying health problems or unmet needs leading to undesirable behaviors. Therefore, the patient’s behaviors due to communication issues should not be misinterpreted and hastily decisions on the use of PRN medications should not be made (Walsh et al., 2020).

Undertaking a formal risk assessment is an important step for making a decision on the use of PRN medications. It consists of the assessment of the risk to the patient themselves, to other patients, and to healthcare providers (Jimu and Doyle, 2019). Relevant assessment tools at the hospital admission determines the need for PRN medication use. For example, the Positive and Negative Syndrome Scale-Excited Component (PANSS-EC) can be used to evaluate the control of agitation and aggression in people with schizophrenia during the first 3 days of admission. Its score influences the decision on the administration of PRN medications (Chaichan, 2008). The study by Chaichan (2008) showed that the mean number of episodes of aggression in patients with schizophrenia during the period of hospitalization was remarkably lower among those assessed with the PANSS-EC. The Positive and Negative Symptom Scale (PANSS) used for the accurate assessment of psychiatric patients with appropriate tools can help determine the need for PRN medications and reduce PRN medication use during hospital stays (Barr et al., 2018). Use of decision support tools for evaluating pain and sedation can optimize PRN medication administration (Procaccini et al., 2020).

In addition to screening tools, the general pattern of the disease, underlying cause of behaviors (Walsh et al., 2020), and underlying diagnosis (Barr et al., 2018) can be helpful for decision-making about PRN medication administration. Nurses have the best position to use their knowledge about patients with long-term health conditions and observe distinctive behavioral patterns and help with the determination of the patient’s needs for PRN medications (Walsh et al., 2020).

Healthcare providers should note that the administration of wrong PRN medications can hamper the diagnosis. Particularly analgesics and antispasmodics can conceal the patient’s symptoms (Di Giulio and Crow, 1997). PRN medication administration should be to the best interest of patients. Healthcare providers should follow a middle ground with regard to how to manage disruptive behaviors using PRN medications without causing medication toxicity (Walsh et al., 2020).

Alternative treatment strategies including non-pharmacologic methods such as redirection and distraction, and physical restraint as the last resort can be considered before the use of PRN medications (Walsh et al., 2020). Restraint, time out, and seclusion can be used to help de-escalation before further PRN medication administration (Usher et al., 2010). Although the use of restrains is outlawed, a balance should be present between the administration of PRN medications and avoiding the use of seclusion, because seclusion is restrictive and has the potential of damaging the therapeutic relationship between healthcare professionals and patients (Barr et al., 2018).

#### 3.4.4 Monitoring and Follow up Interventions

The use and effects of PRN medications should be regularly and systematically evaluated (Baker et al., 2007a). Healthcare providers should be aware of the potential side effects of PRN medications (Baker et al., 2007a) and have a concern about both their ineffectiveness and side effects (Usher et al., 2009). The patient should be regularly checked in terms of physical health before and after PRN medication use (Usher et al., 2010) and probable medication interactions/allergic reactions (Baker et al., 2007a). Side effects of PRN medications can be identified through the monitoring of vital signs and the patient’s symptoms such as extrapyramidal complications (Al-Sughayir, 2014; Al-Sughayir, 2017). In addition, there is a need to assess the effects of PRN medications on the patient’s underlying health condition (Griffiths et al., 2019). The nurse should communicate with the physician as prescriber in case of unsuccessful outcome of PRN medication use (Procaccini et al., 2020). The treating physician should be informed and asked for a medical evaluation in case of any concern regarding the use of PRN medications (Al-Sughayir, 2014; Al-Sughayir, 2017). Assessing the peak time of PRN medication administration during the day can help take appropriate measures to optimize PRN medication use (Akram et al., 2014).

Monitoring and documentation of related data when PRN medications are administered (Al-Sughayir, 2014; Al-Sughayir, 2017; Martin et al., 2018a) and post medication administration are of utmost importance (Akram et al., 2014). Documentation of PRN medications should be clear in terms of the reason (Craven et al., 1987; Martin et al., 2018a) and indication of use (Stein-Parbury et al., 2008), circumstances and symptoms leading to administration (Baker et al., 2007a; Martin et al., 2018a), related effects (Baker et al., 2007a; Curtis et al., 2007; Stein-Parbury et al., 2008; Al-Sughayir, 2014; Al-Sughayir, 2017; Martin et al., 2018a), negative consequences and side effects (Martin et al., 2018a), and the method used for the evaluation of expected outcomes (Curtis et al., 2007). Martin et al. (2018a) in their study reported that in 15% of cases, the administration of psychotropic PRN medications were not documented and in 79% of cases, a reason for it was mentioned. In another study, only in 63.2% of episodes a reason for PRN medication administration was documented (Stein-Parbury et al., 2008). In the study by Curtis et al. (2007), the effect of PRN medications was documented only in 38.8% of occasions.

When PRN medications are used, any additional pre- or post -intervention should be recorded (Curtis et al., 2007). According to the Curtis et al.’s (2007), additional pre- or post -intervention was documented only in 28% of occasions of PRN medication administration. If more than one PRN medications is administered, indications should be clearly explained (Stein-Parbury et al., 2008).

Healthcare providers should take more responsibility for the prescription and administration of PRN medications and should be aware of issues resulting from high doses and polypharmacy especially in patients with mental health problems (Barr et al., 2018; Griffiths et al., 2019; Jimu and Doyle, 2019), and avoid polypharmacy if possible (Al-Sughayir, 2014; Al-Sughayir, 2017).

In those patients who are at the risk of high doses of medications including in long-term care facilities and with severe cognitive issues, the administration of PRN medications and daily recommended dose of PRN medications should be monitored (Sharma et al., 2021). Assessment of over-prescription, over-medication, and polypharmacy of PRN medications should encompass patients with more dependency levels (Stasinopoulos et al., 2018). Monitoring the number of medications in patients with a longer duration of hospital stay is required (Dörks et al., 2016).

#### 3.4.5 Deprescription Strategies

The PRN medication regimen should be completed and its use should be discontinued by the treating physician as soon as possible (Al-Sughayir, 2014; Al-Sughayir, 2017). The end date should be clearly stated at the beginning of prescription (Usher et al., 2009). As a rule, PRN medications should be deprescribed when they are no longer needed (Craven et al., 1987). A time-limited prescription of PRN medications requires the regular review of medication use (Baker et al., 2007a). The use of a stop-order policy after 7 days can help avoid unnecessary PRN medication use and the early deprescription of PRNs. Accordingly, the prescriber has to reassess the PRN medication order and decide on the need or for continuation for more than 7 days. In case of the needs for continuation, the prescriber repeats the order with the consideration of the prescription requirements (Craven et al., 1987). Drug dependency and abuse should be considered when making such a decision (Kaur et al., 2009).

Alternative interventions such as non-pharmacologic strategies on appropriate occasions prior or instead of PRN medication administration or in combination with them not only help achieve an optimal response, but also prepare the ground for discontinuation (Geffen et al., 2002; Usher et al., 2009; Swart et al., 2011; Al-Sughayir, 2014; Al-Sughayir, 2017; Martin et al., 2018a; Barr et al., 2018; Jimu and Doyle, 2019). The feasibility of their use depends on that the patient is identified by healthcare professionals to have a low risk level for use along with having a positive attitude toward such interventions (Usher et al., 2009). Behavior therapy, music, counselling, relaxation, redirection, and planned ignoring have been shown helpful in the reduction of PRN medication use and improvement of self-care (Swart et al., 2011; Al-Sughayir, 2014; Al-Sughayir, 2017; Barr et al., 2018). Combination and the simultaneous use of PRN medications with alternative interventions improve the effectiveness of medication use (Akram et al., 2014; Barr et al., 2018). However, the alternative and non-pharmacologic interventions are time-consuming and their practice requires appropriate expertise (Walsh et al., 2020).

#### 3.4.6 Healthcare Professionals’ Role

Healthcare providers should show their good intentions and positive attitudes toward PRN medication use to be able to perform related caring measures (Edwards et al., 2001). They need to rely on their theoretical and practical knowledge (Di Giulio and Crow, 1997). Having sufficient pharmacotherapeutic knowledge is important for PRN medication use, post-PRN monitoring, and its documentation (Nilsen et al., 2020). Practical experience and knowledge are important and refers to having knowledge about how PRN medications can impact on the patient’s health condition (Nilsen et al., 2020).

Healthcare providers should gain knowledge of any advance directive with regard to PRN medications (Baker et al., 2007a). Appropriate education to healthcare providers can empower them to comply with PRN medication standards such as the dosage guideline (Carder, 2012; Procaccini et al., 2020). Healthcare providers should know about the regulations of PRN medication use in terms of the reason for use, schedule and route, circumstances of use, maximum dose, when to contact the physician, and when to discontinue medications (Carder, 2012). Experienced healthcare providers can teach newly staff regarding the facility-specific systems for PRN medication order, stock, documentation, and administration (Carder, 2012). Personal skills of healthcare providers can contribute to the assessment of the effects of PRN medications (Walsh et al., 2020).

Having access to a senior healthcare provider who is involved in PRN medicines management and discussion on its administration improve medication safety (Jimu and Doyle, 2019). Healthcare providers can seek a second opinion prior to the final decision making regarding PRN medication administration as complementary to their own competence (Nilsen et al., 2020). Sufficient information sharing in both written and oral formats influences PRN medicines management. Quality of the documentation is a significant element in the decision-making process with regard to the PRN medication use. Oral information sharing during shift handoff can inform the next healthcare provider about challenges during the work shift, the patient’s health condition, and how to face issues with PRN medication use (Carder, 2012; Nilsen et al., 2020). Clear, accurate, and up-to-date prescription information avoids uncertainty between the prescriber and the administrator, improves optimal PRN medicines management, and prevents misinterpretations (Usher et al., 2009).

Environmental factors also can influence decisions regarding PRN medication administration. Appropriate staffing on each work shift improves high-quality PRN medicines management (Nilsen et al., 2020). In contrast, staff shortages and heavy workloads increase the inappropriate use of PRN medications (Walsh et al., 2020). When staff shortages are present, healthcare providers are busy and do not have enough time for the patient’s assessment. Therefore, they may give PRN medications more regularly to patients without attempting to take more time and use alternative strategies (Usher et al., 2009; Walsh et al., 2020). Inexperienced healthcare providers who may not be quite familiar with the healthcare setting are the reason for the higher rate of PRN medication use (Usher et al., 2009).

The appropriate storage of PRN medications can facilitate access to medications and is a contributing component of safe PRN medicines management (Carder, 2012; Nilsen et al., 2020). Other aspects are placing medications in a labeled container inside the locked cabinet, adding a direction about conditions in which the medication can be administered, and informing the healthcare provider about the availability of medications (Carder, 2012). Where PRN medications are in storage and a healthcare provider has the key, other healthcare providers have to discuss with her/him and explain the situation before asking for medications, thereby regulate PRN medicines management (Nilsen et al., 2020).

The culture of applying non-pharmacological interventions before administering PRN medications can prevent inappropriate medication use (Nilsen et al., 2020). Clinical protocols where they restrict the physical restraint policy and the use of chemical restraint when non-pharmacological strategies such as distraction and redirection fail to alleviate unfavorable behaviors are supported (Walsh et al., 2020).

Additionally, healthcare professionals’ disciplines or philosophical perspectives regarding the use of PRN medications impact medication use. Some clinical protocols enforce healthcare providers to administer PRN medications or to apply an alternative strategy. However, some healthcare providers may prefer to discuss with their patients and seek alternative strategies and resolve the problem without the use of PRN medications (Usher et al., 2009). On the other hand, some healthcare providers may use PRN medications to facilitate patients’ adjustment to the requirement of the work environment during hospitalization (Walsh et al., 2020).

#### 3.4.7 Participation of Patients and Families

Positive attitudes of the patient and his/her family members toward PRN medications influence PRN medicines management (Edwards et al., 2001). The patient’s preference and compliance with PRN medication use influence healthcare provider’s decisions on the administration of PRN medications (Di Giulio and Crow, 1997; Walsh et al., 2020). The patients’ involvement is a substantial aspect of the decision process for PRN medication use (Nilsen et al., 2020). If the patient can reliably express his/her symptoms and request for medications, it is easiest for healthcare providers to decide about PRN medication use (Carder, 2012; Akram et al., 2014). However, patients’ self-request for PRN medications may not be completely to their best interest, specifically for medications that increase the risk of dependence and abuse (Akram et al., 2014). Wherever possible, joint decision-making about the prescription of PRN medications is recommended on translating/agreeing the rational/indication for the prescription into the language of/with the patient (Baker et al., 2007a).

The patients’ knowledge regarding their medications also is important. For instance, when healthcare providers improve their patients’ knowledge of the side effects of a particular PRN medication, it is more likely that the patient accepts non-administration of medications (Nilsen et al., 2020). The patient’s willingness can influence the replacement of medications with alternative and non-pharmacological interventions (Usher et al., 2009).

Communication and cognitive abilities of the patient to assess the necessity of PRN medication use also have been emphasized. For example, the patient’s wellness informs healthcare providers of the patient’s ability to convey the situation that raises the need for the administration of PRN medications (Nilsen et al., 2020). The patient’s ability to cooperate may be influenced by the administration of some PRN medications such as analgesics (Di Giulio and Crow, 1997). Conflicting understanding between healthcare providers and family members regarding the patient’s need for PRN medications should be resolved through reaching agreement by all parties involved in patient care (Nilsen et al., 2020).

When the patient looks unwell and for instance expresses the signs of aggression, agitation, or elated mood, PRN medication use is more likely (Usher et al., 2009). A direct association has been shown between the severity of symptoms and the dose of PRN medications (Griffiths et al., 2019).

For outpatients and those who have to manage PRN medications themselves, applying some instructions and strategies on PRN medication bottles under the name of the Take-Wait-Stop label design is beneficial. Deconstructing prescription wording about PRN instructions can explicitly convey the dose, interval between doses, and maximum daily dose to patients and their families. It consists of explicit, deconstructed instructions, and simplified texts such as numeric characters instead of words, e.g., “1 tab” instead of “one tab,” and carriage returns to place each part of the instructions on separate lines. The use of simplified text and plain language, “Stop” to replace the typical wording “do not exceed,” can inform patients with limited literacy levels about the maximum daily dose (McCarthy et al., 2013; McCarthy et al., 2019).

#### 3.4.8 Multidisciplinary Collaboration

Collaboration by healthcare professionals is needed from the moment that PRN medications are prescribed. Nurses spent the most time with patients and have the central role for identifying the patient’s need for PRN medications. Physician’s and nurse’s collaboration regarding PRN medication use has been emphasized (Jimu and Doyle, 2019). Interprofessional medication review with the collaboration of pharmacist on the patient’s medication list facilitates updating medications and changing and removing unused ones. It also creates consensus on PRN medication use (Nilsen et al., 2020). Also, involvement of the multidisciplinary team in the management of patients’ behaviors using alternative methods reduces the need for PRN medication use (Barr et al., 2018).

## 4 Discussion

In this systematic review with an integrative approach, the practical considerations of PRN medicines management were suggested. They can help with the improvement of quality and safety of the PRN medication process. Our review findings showed the need for appropriate assessment and planning for safe PRN medication use and inclusion of strategies for the improvement of multidisciplinary collaboration, monitoring of medications’ effects and side effects, deprescription, use of alternative therapies, and involvement of patients and families in medication therapy.

Healthcare professionals’ collaboration for making decisions on the prescription and administration of PRN medications is important. For instance, double-checking by at least two healthcare providers can prevent medication errors (Koyama et al., 2020; Vaismoradi et al., 2020). However, the role of electronic and digital solutions for improving the safety of PRN medicines management has remained unattended. Electronic prescribing and administrating of medications have the potential for reducing the risk of medication errors and adverse drug events (Ammenwerth et al., 2008; Slight et al., 2019). A systematic review and meta-analysis reported a considerable (50%) reduction in preventable adverse drug events when electronic prescribing systems in acute care settings were used in healthcare settings (Nuckols et al., 2014). It can ensure the safety of PRN prescribing through the provision of important capabilities such as decision support, specification of indications for the PRN medication use and the maximum daily dose, provision of appropriate alert, and communication between prescribers and administrators (Donyai et al., 2008; Baysari et al., 2012; Martin et al., 2017).

Since the accurate documentation of patient information is one of the primary competences of healthcare providers and facilities the monitoring of PRN medications, structured report templates regardless of the method of documentation can improve PRN medication documentation (Hammer et al., 2019). The electronic health record with the inclusion of information about effectiveness, side effects, and matching between the indication of PRN prescription and administration contributes to the high quality documentation process (Martin et al., 2017).

In addition, the significance of assessment tools regarding the effectiveness of PRN medication use was not acknowledged in the included studies to this review. In an instrument development study, Silk et al. (2013) suggested that the provision of an accurate evaluation of the effectiveness of PRN medications as a result of decreased subjective and ambiguous language improved the patient outcomes (Silk et al., 2013). The prevention of polypharmacy along with PRN medication use requires appropriate screening tools. Although such a specific tool is not available yet, the STOPP (screening tool of older persons’ potentially inappropriate prescriptions) and START (screening tool to alert doctors to the right treatment) tools can be used to review medications for vulnerable people and identify potentially inappropriate medications (O'Mahony et al., 2015; Vaismoradi et al., 2020).

Our review findings highlighted the deprescription of PRN medications and its replacement with non-pharmacologic methods to prevent polypharmacy and medication abuse. The plan for deprescribing process of PRN medications should be devised based on each patient’s need and under close monitoring (Renn et al., 2018; Vaismoradi et al., 2021a). Also, cost and benefit assessment with regard to the continuation and discontinuation of medications should be performed (Renn et al., 2018). Moreover, the concerns of patients and their informal caregivers about the replacement of medications by alternative therapies that can influence their collaboration with the deprescription plan should be taken seriously (Scott et al., 2015; Vaismoradi et al., 2021a). Given that the use of PRN medications reduce the inclusion of other therapeutic interventions in the therapeutic plan (Hipp et al., 2018), PRN medications should not be used when potential non-pharmacological treatment options are available (Martin et al., 2017).

According to our review findings, healthcare professionals’ competencies for PRN medicines management influenced the safety of the medication process. Their pharmacological competence as having sufficient knowledge and skills to manage real-life medication circumstances and making appropriate decisions (Sulosaari et al., 2011; Salehi et al., 2021) are affected by the complexity of the patient’s medication processes (Sulosaari et al., 2011; Lichtner et al., 2016). Healthcare providers need education and training about the application of alternative and non-pharmacological interventions for relieving patients’ symptoms (Molloy et al., 2012). They should be educated to avoid overreliance on PRN medications (Zeisel et al., 2016; Harper et al., 2017; Martin et al., 2018b).

Patient participation and shared decision-making was a pilar for safe PRN medicines management in our review. Patients play an active role in care planning and should have the opportunity to participate in decision making (Mikesell et al., 2016). Patients eagerly participate in decision-making if they receive sufficient knowledge about their medications, have appropriate understanding of PRN medications (Hipp et al., 2018), and are able to define PRN medications and the rational for their use (Morkunas et al., 2016). It also enhances their compliance to the medication regimen (Fernandez et al., 2006; Mardani et al., 2020). Therefore, the opportunity for asking about PRN medications and giving consent when PRN medications are offered should be given to patients (Hipp et al., 2018; Vaismoradi et al., 2021b).

### 4.1 Strengths and Limitations

This systematic review using international databases can improve our understanding of practical considerations that should be applied by healthcare professionals for safe PRN medicines management. We identified relevant literature with qualitative and quantitative research designs by applying multidimensional keywords for a systematic search on international databases. Therefore, our findings provide an extensive overview of the present international knowledge regarding this important clinical topic. However, our review was limited to studies published in the English language due to restriction in translation. Future studies need to consider grey literature and other sources of literature including local guidelines used in clinical settings and in other languages to improve the generatability of our review findings. Also, the majority of retrieved studies in the present systematic review was from Australia, the United States, Canada, and European countries. A limited number of studies from Asia and Africa on PRN medicines management was retrieved. Therefore, PRN medicines management should be addressed in other research contexts to improve our understanding of cultural aspects affecting medication safety.

## 5 Conclusion

The current review sought to summarize and integrate practical considerations by healthcare professionals for PRN medicines management in different healthcare settings. The findings of this review demonstrate that PRN medicines management is a complex process and many factors influence its safety. We identified a range of possible practical measures that should be taken for improving the safety of PRN medication therapy.

The synthesised knowledge in our review can be used to develop optimal PRN medicines management guidelines in different clinical settings and to investigate its effect on safe care indicators. A suggested list of practical considerations for PRN medicines management has been developed based on our review findings and has been presented in Table 4. After making it suitable for application in clinical practice, they can be used to guide healthcare professionals in PRN medicines management situations. Along with other medication safety measures, the suggested implications can support healthcare practitioners’ decision making for improving the quality and safety of PRN medication use.

**TABLE 4:** The suggested list of the practical considerations of PRN medicines management.

Category	Item
PRN indications and precautionary measures	Prescription based on the diagnosis and the assessment of the patient and his/her medical history
	Specification of appropriate indications and the purpose of medication use
	Consideration of the efficacy and side effects of new atypic medications
	Attention to clinical indications for the continuation or discontinuation of medications
	Replacement of PRN medications by regular medications with suitable doses
Requirements of PRN prescription	Medication reconciliation immediately after admission
	Documentation of the medication name, dose, route of administration, and the physician’s instructions
	Inclusion of prescription details such as the reason for use, shortest time allowed before dose repetition, time intervals, maximum dose per 24 h, and sequencing PRN medications for the same healthcare problem
	Consideration of the patient’s preferences in the prescription of medications
	Setting undesirable patients’ behaviors and ineffectiveness of non-pharmacological methods as legitimate reasons for medication prescription
	Prioritizing oral medications to injections when the required response can be achieved
Interventions for PRN administration	Setting clear goals and having ration underpinning medication administration
	Administration of rapid tranquilizations along with logic and reasons
	Setting concordance between the indication of prescription and administration of medications
	Involvement of patients and informal caregivers through informing them about the rationale of PRN medication use, related perceived risks, and seeking consent before medication administration
	Avoiding the violation of the minimum time specified between doses
	Selection of the best route for medication administration
	Medication administration when there is the risk of patient harm
	Making decisions on medication administration after thorough assessment of patients and related health history
	Interviewing and observation of the patient before medication use
	Interpretation of the patient’s actions and non-verbal clues
	Consideration of the severity of the patient’s health condition and related symptoms
	Collective decision-making based on collected data and personal judgments by all healthcare providers
	Incorporation of probable risks into the indications of medication administration
	Going beyond problem-specific symptoms for medication administration
	Avoiding the misinterpretation of the patient’s behaviors and taking hastily decisions
	Risk assessment for decision making on medication administration
	Prevention of administration of medications that cause toxicity and hamper diagnosis
	Administration of medications in the best interest of patients
	Use of alternative and non-pharmacologic methods before medication administration
	Use of restraint, time out, and seclusion to help with de-escalation before medication administration
Monitoring and follow up interventions	Regular and systematic evaluation of the effects of medications on the symptoms of the underlying health condition
	Being aware of the potential side effects of PRN medications and having concerns about their ineffectiveness and side effects
	Regular checking of the patient’s physical health and probable medication interactions/allergic reactions before and after medication use
	Communication of the unsuccessful outcome of PRN medication use and any concern to the prescriber
	Assessing the peak time of daily medication use to take appropriate measures for medication optimization
	Detailed documentation of the medication procedure in terms of indication for use, circumstances and symptoms leading to administration, related effect, negative consequences and side effects, and methods used for expected outcomes’ evaluation
	Knowledge improvement about issues resulting from high doses and polypharmacy
	Close monitoring of medications use in patients who are at the risk of polypharmacy, dependency, overdose and showing allergic reactions
	Monitoring of the number of medications in patients with a longer duration of hospitalization
Deprescription strategies	Completing the medication regimen and its early discontinuation
	Determining the end date for medication use at the beginning of prescription
	Time-limited prescription of PRN medications using a regular review
	The use of a stop-order policy after 7 days to avoid unnecessary medication use
	Consideration of drug dependency and abuse to make deprescription decision
	Use of alternative and non-pharmacologic methods on appropriate occasions instead of medications or in combination
Healthcare professionals’ role	Appropriate individualized philosophical perspectives and positive attitudes toward medication use
	Improving theoretical and practical knowledge of medicines management
	Education of healthcare staff to comply with standard medication use
	Education of new staff by experienced and senior ones with regard to medication order, stock, documentation, and administration
	Seeking a second and expert opinion prior to medication administration
	Sharing information between healthcare providers in both written and oral formats regarding PRN medicines management
	Clear, accurate, and up-to-date information sharing to avoid ambiguity between the prescriber and administrator
	Appropriate staffing pattern on each work shift for medication administration
	Appropriate storage of medication, e.g., in a labelled container inside the locked cabinet and direction regarding the conditions in which the medication can be administered
	Establishing the culture of non-pharmacological interventions before medication use
	Use of medications to facilitate patients’ adjustment to the requirements of the work environment during hospitalization
Participation of patients and families	Creating positive attitudes in the patient and informal caregivers about medication use
	Attention to the patient’s preferences and compliance with medication use
	Involvement of the patient in the decision process for medication use
	Joint decision-making about the prescription of medications and translating/agreeing the rational/indication into the patient’s language
	Improvement of the patients’ knowledge regarding the medication process
	Encouraging the patient to replace medications with alternative and non-pharmacological methods
	Resolving conflicting understanding of medication use between healthcare providers, patients, and informal caregivers
	Connecting the severity of symptoms and medication doses
	Use of instructions on the medication bottles under the name of the Take-Wait-Stop label for outpatient and ambulatory patients
Multidisciplinary collaboration	Collaboration by healthcare professionals from the moment that PRN medications are prescribed
	Identifying and highlighting nurses’ roles for medicines management
	Interprofessional medication review on the patient’s medication list to reach consensus on medication use
	Involvement of the multidisciplinary team in the management of patients’ behavioral problems

It should be acknowledged that alternative interventions such as non-pharmacologic strategies in appropriate caring occasions have priority over PRN medication use due to fewer side effects. Therefore, healthcare providers should improve their competencies to avoid overreliance on PRN medication use for relieving patients’ symptoms.

## Data Availability

The original contributions presented in the study are included in the article/Supplementary Material, further inquiries can be directed to the corresponding author.
