# Supplementary material for: Practical Considerations of PRN Medicines Management: An Integrative Systematic Review
Source: Front Pharmacol. 2022 Apr 12;13:759998. doi: 10.3389/fphar.2022.759998 (PMC9039188; doi:10.3389/fphar.2022.759998)
Supplement: Supplementary file 1 [file DataSheet1.docx]

**Supplementary file 1**. The protocol for the systematic review of the practical considerations of PRN medicines management

| **Review title** | Practical considerations of PRN medicines management: An integrative systematic review |
| --- | --- |
| **PICO (population, intervention/Index, control, and outcomes) statement** | P (Population): healthcare providers including nurses, physicians, and pharmacists involved in PRN medicines management;  I (Interest): practical considerations in terms of interventions and strategies by healthcare professionals for prescription, dispensing, administration, monitoring, and deprescription of PRN medicines;  Co (Context): all contexts in healthcare consisting of child, adult, physical and mental health. |
| **Anticipated or actual start date** | 01/01/2021 |
| **Anticipated completion date** | 30/06/2021 |
| **Review team details and rolls** | This review is conducted by a team of international researchers including nurse (MV, AM, PP), physician (CW), and pharmacist (SJ) who have sufficient knowledge and expertise on medicines management and systematic reviews. The research team consists of the following researchers:  Abbas Mardani, PhD Candidate in Nursing, Iran University of Medical Sciences, Tehran, Iran;  Piret Paal, Institute for Nursing Science and Practice, Paracelsus Medical University, Salzburg, Austria;  Christiane Weck, Paracelsus Medical University, Salzburg, Austria and Department of Neurology, Klinikum Agatharied, Hausham, Germany;  Shazia Jamshed, Faculty of Pharmacy, University Sultan Zainal Abidin, Terengganu, Malaysia;  Mojtaba Vaismoradi, Faculty of Nursing and Health Sciences, Nord University, Bodø, Norway;  The authors contributed to the design and implementation of the research, to the analysis of the results and to the writing of the manuscript as follows; M.V.: Conceptualization; A.M. & M.V.: Data curation, Formal analysis, Investigation, Methodology; Project administration, Resources, Software; A.M. & M.V.: Writing—original draft, Writing-review and editing; A.M., M.V., P.P., C.W. & S.J. All authors have read and agreed to the published version of the manuscript. |
| **Funding sources/sponsors** | This research received no external funding. |
| **Conflicts of interest** | The authors declare that the research is conducted in the absence of any commercial or financial relationships that could be construed as a potential conflict of interest. |
| **Aim of study** | This review aims to identify the practical considerations of PRN medicines management in healthcare settings. |
| **Identification of the research question** | What are the practical considerations/interventions/aspects of PRN medicines management taken by healthcare professionals including nurses, pharmacists, and physicians for PRN medicines management in terms of prescription, administration, and follow up in short-term, long-term, and acute healthcare settings? |
| **Searches** | The online databases of Web of Knowledge, PubMed (including MEDLINE), Cinahl, and Scopus are searched to retrieve empirical studies published by peer-reviewed scientific journals up to end of May 2021.  A librarian at Nord University assists with the search process. |
| **Language** | English |
| **Keywords** | The following keywords using the Boolean method are used for conducting the search:  (PRN OR “pro re nata” OR “as needed” OR “as required”)  AND  (guideline OR “practice guideline” OR “clinical practice guideline” OR “clinical guideline” OR “critical pathway” OR “clinical pathway” OR “critical path” OR “clinical path” OR “patient care planning” OR instruction OR technique OR program*)  AND  (medication OR drug OR medicines OR “pharmaceutical preparations” OR pharmaceuticals OR “medicines management”). |
| **Eligibility criteria** | Inclusion criteria:  Original research studies with qualitative, quantitative and mixed methods designs that focus on PRN medicines management;  Presentation of practical considerations in terms of interventions and strategies for prescription, administration, monitoring, and management of the side effects and adverse drug reactions (ADRs) of PRN medications;  Published in peer-reviewed scientific journals.  Exclusion criteria:  Studies on the management of routinely prescribed medications;  Gray literature including governmental or organizational reports, conference proceedings, doctoral dissertations, mater degree theses, books, and unpublished data. |
| **Condition or domain being studied** | This systematic review presented a synthesis of the current knowledge about PRN medicines management by healthcare providers in healthcare settings. |
| **Search strategy** | The above-mentioned databases are searched for retrieving original studies published up to end of May 2021. Further, a manual search in the references lists of selected studies is performed to improve the search coverage. |
| **Data extraction (selection and coding)** | For data extraction, a table is developed comprising the following sections: (a) the first author’s surname, publication year, and the country where the study was conducted; (b) study design, sample size, and setting; (c) data relating to the practical considerations of PRN medicines management; (d) name and dose of PRN medications and patients’ age group; and (e) healthcare providers involved in PRN medicines management. The accuracy of gathered data before the research synthesis is assessed by the research team through double checking. |
| **Risk of bias and quality assessment** | Two review authors (AM, MV) independently assess the quality and risk of bias in included studies using the following tools:  Quality of the selected studies is evaluated in terms of the appropriateness of research structure and reporting using the Enhancing the Quality and Transparency of Health Research (EQUATOR).  According to the studies’ designs, the following tools are used: (a) the Strengthening the Reporting of Observational Studies in Epidemiology (STROBE) for observational and cross-sectional studies, (b) the Standards for Reporting Qualitative Research (SRQR) for qualitative research, (c) Consolidated Standards of Reporting Trials (CONSORT) for experimental and quasi-experimental studies, (d) the Good Reporting of A Mixed Methods Study (GRAMMS) for mixed-methods studies.  The Cochrane Collaboration’s tool for assessing the risk of bias for randomized clinical trials, the Risk of Bias in Non-randomized Studies of Interventions (ROBINS-I), and the risk of bias assessment for cross-sectional studies adapted from the Newcastle-Ottawa Quality Assessment Scale are used for the assessment of the risk of bias of in selected studies. |
| **Strategy for data synthesis** | The findings of the included studies are reviewed and based on diversities and similarities in their findings, appropriate categories are developed. |
| **Equator guidelines** | The review process will be informed by The Preferred Reporting Items Systematic Reviews and Meta-analysis (PRISMA): http://prisma-statement.org/prismastatement/Checklist.aspx |

**Supplementary file 2**. PRISMA 2020 checklist

| **Section and Topic** | **Item #** | **Checklist item** | **Location where item is reported** |
| --- | --- | --- | --- |
| **TITLE** | | |  |
| Title | 1 | Identify the report as a systematic review. | 1 |
| **ABSTRACT** | | |  |
| Abstract | 2 | See the PRISMA 2020 for Abstracts checklist. | 1-2 |
| **INTRODUCTION** | | |  |
| Rationale | 3 | Describe the rationale for the review in the context of existing knowledge. | 2-3 |
| Objectives | 4 | Provide an explicit statement of the objective(s) or question(s) the review addresses. | 3 |
| **METHODS** | | |  |
| Eligibility criteria | 5 | Specify the inclusion and exclusion criteria for the review and how studies were grouped for the syntheses. | 4 |
| Information sources | 6 | Specify all databases, registers, websites, organisations, reference lists and other sources searched or consulted to identify studies. Specify the date when each source was last searched or consulted. | 4 |
| Search strategy | 7 | Present the full search strategies for all databases, registers and websites, including any filters and limits used. | 4 |
| Selection process | 8 | Specify the methods used to decide whether a study met the inclusion criteria of the review, including how many reviewers screened each record and each report retrieved, whether they worked independently, and if applicable, details of automation tools used in the process. | 4 |
| Data collection process | 9 | Specify the methods used to collect data from reports, including how many reviewers collected data from each report, whether they worked independently, any processes for obtaining or confirming data from study investigators, and if applicable, details of automation tools used in the process. | 5 |
| Data items | 10a | List and define all outcomes for which data were sought. Specify whether all results that were compatible with each outcome domain in each study were sought (e.g. for all measures, time points, analyses), and if not, the methods used to decide which results to collect. | 5 |
|  | 10b | List and define all other variables for which data were sought (e.g. participant and intervention characteristics, funding sources). Describe any assumptions made about any missing or unclear information. | 5 |
| Study risk of bias assessment | 11 | Specify the methods used to assess risk of bias in the included studies, including details of the tool(s) used, how many reviewers assessed each study and whether they worked independently, and if applicable, details of automation tools used in the process. | 4-5 |
| Effect measures | 12 | Specify for each outcome the effect measure(s) (e.g. risk ratio, mean difference) used in the synthesis or presentation of results. | NA |
| Synthesis methods | 13a | Describe the processes used to decide which studies were eligible for each synthesis (e.g. tabulating the study intervention characteristics and comparing against the planned groups for each synthesis (item #5)). | 5 |
|  | 13b | Describe any methods required to prepare the data for presentation or synthesis, such as handling of missing summary statistics, or data conversions. | 5 |
|  | 13c | Describe any methods used to tabulate or visually display results of individual studies and syntheses. | 5 |
|  | 13d | Describe any methods used to synthesize results and provide a rationale for the choice(s). If meta-analysis was performed, describe the model(s), method(s) to identify the presence and extent of statistical heterogeneity, and software package(s) used. | 5 |
|  | 13e | Describe any methods used to explore possible causes of heterogeneity among study results (e.g. subgroup analysis, meta-regression). | NA |
|  | 13f | Describe any sensitivity analyses conducted to assess robustness of the synthesized results. | NA |
| Reporting bias assessment | 14 | Describe any methods used to assess risk of bias due to missing results in a synthesis (arising from reporting biases). | 4-5 |
| Certainty assessment | 15 | Describe any methods used to assess certainty (or confidence) in the body of evidence for an outcome. | 4-5 |
| **RESULTS** | | |  |
| Study selection | 16a | Describe the results of the search and selection process, from the number of records identified in the search to the number of studies included in the review, ideally using a flow diagram. | 5, 45 (Figure 1) |
|  | 16b | Cite studies that might appear to meet the inclusion criteria, but which were excluded, and explain why they were excluded. | NA |
| Study characteristics | 17 | Cite each included study and present its characteristics. | 6, 24-34 (Table 2) |
| Risk of bias in studies | 18 | Present assessments of risk of bias for each included study. | 5, 6; supplementary Figure 1, 2 , 3 |
| Results of individual studies | 19 | For all outcomes, present, for each study: (a) summary statistics for each group (where appropriate) and (b) an effect estimate and its precision (e.g. confidence/credible interval), ideally using structured tables or plots. | NA |
| Results of syntheses | 20a | For each synthesis, briefly summarise the characteristics and risk of bias among contributing studies. | 5-12 |
|  | 20b | Present results of all statistical syntheses conducted. If meta-analysis was done, present for each the summary estimate and its precision (e.g. confidence/credible interval) and measures of statistical heterogeneity. If comparing groups, describe the direction of the effect. | NA |
|  | 20c | Present results of all investigations of possible causes of heterogeneity among study results. | NA |
|  | 20d | Present results of all sensitivity analyses conducted to assess the robustness of the synthesized results. | NA |
| Reporting biases | 21 | Present assessments of risk of bias due to missing results (arising from reporting biases) for each synthesis assessed. | 5, 6; supplementary Figure 1, 2 , 3 |
| Certainty of evidence | 22 | Present assessments of certainty (or confidence) in the body of evidence for each outcome assessed. | 5, 6; supplementary Figure 1, 2 , 3 |
| **DISCUSSION** | | |  |
| Discussion | 23a | Provide a general interpretation of the results in the context of other evidence. | 12-14 |
|  | 23b | Discuss any limitations of the evidence included in the review. | 14 |
|  | 23c | Discuss any limitations of the review processes used. | 14 |
|  | 23d | Discuss implications of the results for practice, policy, and future research. | 14 |
| **OTHER INFORMATION** | | |  |
| Registration and protocol | 24a | Provide registration information for the review, including register name and registration number, or state that the review was not registered. | 3, Supplementary file 1 |
|  | 24b | Indicate where the review protocol can be accessed, or state that a protocol was not prepared. |  |
|  | 24c | Describe and explain any amendments to information provided at registration or in the protocol. | NA |
| Support | 25 | Describe sources of financial or non-financial support for the review, and the role of the funders or sponsors in the review. | 15 |
| Competing interests | 26 | Declare any competing interests of review authors. | 15 |
| Availability of data, code and other materials | 27 | Report which of the following are publicly available and where they can be found: template data collection forms; data extracted from included studies; data used for all analyses; analytic code; any other materials used in the review. | 15 |

**Supplementary file 3**

Figure 1. Risk of bias in randomized controlled trial studies.

A:

| Author, year | A | B | C | D | E | F |
| --- | --- | --- | --- | --- | --- | --- |
| McCarthy et al. (2013) | 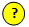 | 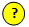 | 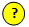 | 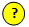 | 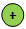 | 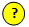 |
| McCarthy et al. (2019) | 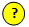 | 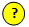 | 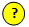 | 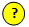 | 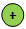 | 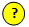 |
| 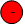 Yes (high risk of bias) 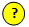 Unclear 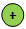 No (low risk of bias)  (A) Bias in random sequence generation (Selection bias)  (B) Bias in allocation concealment (Selection bias)  (C) Bias in the blinding of participants and personnel (Performance bias)  (D) Bias in the blinding of outcome assessment (Detection bias)  (E) Bias in incomplete outcome data (Attrition bias)  (F) Bias in selective outcome reporting (Reporting bias) | | | | | | |

B:

Figure 2. Risk of bias in non-randomized studies.

A:

| Author, year | A | B | C | D | E | F | G |
| --- | --- | --- | --- | --- | --- | --- | --- |
| Edwards et al. (2001) | 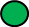 | 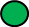 |  | 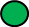 |  |  |  |
| Procaccini et al. (2020) |  |  | 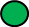 | 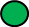 | 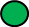 |  |  |
| 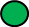  Low risk of bias Moderate risk of bias  Serious risk of bias Critical risk of bias  No information  (A) Bias due to confounding (Confounding)  (B) Bias in the selection of participants into the study (Selection bias)  (C) Bias in the classification of interventions (Information bias)  (D) Bias due to deviations from intended interventions (Confounding)  (E) Bias due to missing data (Selection bias)  (F) Bias in the measurement of outcome (Information bias)  (G) Bias in the selection of reported result (Reporting bias) | | | | | | | |

B:

Figure 3**.** Risk of bias in cross-sectional studies.

A:

| Author (year) | Bias in the assessment of exposure | Bias in the development of the outcome of interest in case and controls | Bias in the selection of cases | Bias in the selection of controls | Bias in the control of prognostic variable |
| --- | --- | --- | --- | --- | --- |
| Craven et al. (1987) |  |  |  |  | 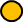 |
| Di Giulio and Crow (1997) |  |  |  |  |  |
| Geffen et al. (2002) |  |  |  |  |  |
| Baker et al. (2007a) |  |  |  |  |  |
| Curtis et al. (2007) |  |  |  |  |  |
| Chaichan (2008) |  |  |  |  |  |
| Gordon et al. (2008) |  | 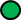 | 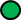 | 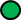 |  |
| Stein-Parbury et al. (2008) |  |  |  |  |  |
| Kaur et al. (2009) |  | 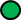 |  |  | 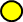 |
| Mullen and Drinkwater (2011) | 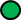 | 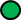 |  |  | 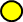 |
| Swart et al. (2011) |  |  |  |  | 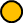 |
| Akram et al. (2014) |  |  |  |  | 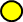 |
| Al-Sughayir (2014) |  |  |  |  |  |
| Russell et al. (2014) |  |  |  |  | 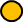 |
| Dörks et al. (2016) |  |  |  |  |  |
| Al‑Sughayir (2017) |  |  |  |  |  |
| Barr et al. (2018) |  |  |  |  |  |
| Stasinopoulos et al. (2018) |  |  | 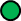 | 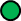 |  |
| Griffiths et al. (2019) |  |  |  |  |  |
| Sharma et al. (2021) |  |  |  |  |  |
| Definitely No (low risk of bias) Probably no (Probably low risk of bias)  Definitely yes (high risk of bias) Probably Yes (Probably high risk of bias) | | | | | |

B:

**Supplementary file 4**. Initial list (provisional) of items and categories of the practical considerations of PRN medicines management in healthcare settings.

| **Item** | **Category** |
| --- | --- |
| - Specifying the indication for PRN prescriptions  - Description of the Clear purpose for PRN medications  - Description of indications for the prescription of PRN medications  - Having concerns about the prescription of the new atypical medications as PRN  - Consideration of clinical indications for the use or discontinuation of PRN medications  - Consideration of the underlying diagnosis for the prescription of PRN medications  - Prescription of regular medications instead of PRN medications  - Use of regular medications as PRN for individual patients  - Prescription based on the thorough assessment of patients and his/her background | PRN indications and precautionary measures |
| - Application of specifically designed sheets for PRN medicines management containing medication name, dose, route of administration, and an empty space for the physician's instructions  - Stating the time interval between PRN doses and maximum dosage limit per 24 hours during medication prescriptions  - Inclusion of the name of the medication, dose, route of administration, reason for use, and shortest time allowed before, and repetition of the dose in the physician order  - Sequencing PRN medications for the same healthcare problem  - Use of fast acting medications to prevent patient’s self-harm  - Control of undesirable behaviors to legitimate PRN medication use  - Use of PRN medications to reduce agitation in patients who are unable to follow their previous habits such as smoke cigarette, drink alcohol or access to illicit drugs  - Use of oral PRN medications when the patient accepts and when the required response is achieved rather than injections  - Reconciliation and documentation of current medications after admission | Requirements of PRN prescription |
| - Making decision based on collected data for the administration of PRN medications  - Perceived patient’s harm and the probability of risk by healthcare providers before PRN medication administration  - Consideration of patient’s symptoms and behaviors  - Consideration of laboratory test results before medication administration  - Data collection on vital signs before medication administration  - Having a closer look at psychological symptoms with a broad perspective rather than being problem-oriented  - Being worried about the administration of wrong medications that can hamper diagnosis  - Use of both subjective (internal state) and objective (behavior) assessment methods to make decisions on medication use  - Indication of a clear purpose for PRN medication administration  - Congruence between the indication of prescription and administration  - Assessment of allergies prior to medication administration  - Having clear goals underpinning the administration of PRN medications  - Application of assessment tools during admission to determine the need for PRN medications  - Consideration of sedation level, pain intensity, respiratory rate, and prior response for the administration of opioids;  - Consideration of the patient’s behavior, concerns, and requests  - Close monitoring of the patient’s behaviors and symptoms to find the indication of medication administration  - Use of nonpharmacologic strategies such as restrain before medication use  - Collective decision making on PRN medications based on the nurse’s perspectives and the patient’s behaviors and symptoms  - Use of PRN medications based on the prediction of the pattern of patient’s behaviors  - Consideration of the disease’s general pattern and the underlying cause of behaviors  - Use of decision support tools before medication administration  - Assessing the patient’s physical and psychological symptoms  - Undertaking risk assessment with regard to harm to the patient and others around them  - Preparing the patient about the timing of PRN medication administration  - Consideration of underlying diagnosis for PRN medication administration  - Attention to the patient’s request before medication administration  - Accurate assessment and the use of appropriate tools to determine the need for PRN medication administration  - Assessing the reason for PRN medication use as rapid tranquilization  - Interpretation of the patient’s nonverbal behavioral clues such as gesture as reason for PRN medication administration  - Regular patient’s checking in terms of physical health before medication use  - Deescalation through restraints and seclusion before PRN medication administration  - Not supporting the medication request by patients with drug-seeking habits  - Administration based on the thorough assessment of patients and his/her background  - Communicating the rational for medication administration and any perceived risks, answering questions and seeking consent from the patient  - Severity of the patient’s health condition and symptoms as the factor affecting medication administration  - Interpretation of the patients’ symptoms for PRN medication use  - Administration of PRN medications to the best interest of the patient  - Use of PRN medications to reduce the use of seclusion and restrictive measures damaging therapeutic relationships  - Misinterpretation of the patient’s behaviors due to communication issues and use of PRN medications  - Avoiding PRN administration when the minimum time specified between the doses of medications are violated  - Paying attention to the interval and dose of the re-administration of similar PRN medications  - Planning for the timing and administration route of PRN medications | Interventions for PRN administration |
| - Regular and systematic evaluation of the effects of PRN medications for individual patients  - Communication with the prescriber in case of the unsuccessful outcome of PRN  - Evaluation of the effects of PRN medications on symptoms and underlying health conditions  - Monitoring vital signs for side effects, such as extrapyramidal, of PRN medication administration  - Informing the treating psychiatrist and asking for a medical evaluation in case of any concern  - Regular patient’s checking in terms of physical health before and after medication use  - Being aware of the potential side effects of PRN medication  - Concerns about ineffectiveness and side effects of medications  - Recording the reason for PRN medication administration  - Documentation of circumstances leading to the administration of PRN medications and its beneficial or detrimental effects on the patient’s behavior  - Description of the method used for the evaluation of PRN medication effects  - Documentation of any additional pre- or post-intervention when PRN medications are used  - Documentation of the administration of more than one PRN medications  - Documentation of the outcome of PRN medication administration  - Identification and documentation of symptoms related to the need for PRN medication use  - Documentation of PRN medications when it is administered  - Documentation of the reason for PRN medication administration  - Documentation of the effect and side effect of PRN medications  - Documentation of the patient’s response to medication  - Recording the post-administration monitoring process  - Assessment of the daily dose recommendation of medications;  - Monitoring PRN medication administration in patients with severe cognitive issues  - Assessment of over-medication and polypharmacy;  - Assessment of over-prescription of PRN medications in patients with more dependency levels  - Taking more responsibility in prescribing and administering PRN medications and being aware of issues resulting from high dose and poly-pharmacy  - Monitoring the number of medications in patients with a long duration of hospitalization  - Monitoring side effects and additional medication interactions/allergic reactions  - Assessing the peak time of medication administration in the day | Monitoring and follow up interventions |
| - Use of stop-order policy after 7 days, and reassessment of more prescriptions by the physician  - Deprescribing PRN medications when they are no longer needed  - Time‐limited prescription of PRN medications along with regular reviews  - Completing the PRN regimen order among the treating psychiatrist as soon as possible  - Consideration and use of alternative interventions instead of PRN medications  - Trying non-pharmacological interventions prior to the administration of PRN medications  - Use of alternative methods such as music and relaxation to reduce PRN use  - Consideration of alternative methods (e.g., counselling) when handling the patient’s difficult behaviors before resorting to PRN medications  - Assessing techniques to be used for preventing PRN medication use including counselling, prompt to calm, redirection, planned ignoring, offering alternative choices, and reminder of consequences  - Combination of PRN medications with other methods to improve their effectiveness  - Time-consuming identity of nonpharmacologic interventions such as distraction and redirection  - Use of alternative methods such as behavior therapy and relaxation instead of PRN to improve self-care  - Simultaneous use of PRN medications and restrains  - Prevention of drug dependency and abuse | Deprescription strategies |
| - Relying on theoretical and practical knowledge for PRN medication administration  - Having a good intention to administer PRN medications  - Positive attitudes by healthcare providers toward PRN medication use  - Having the ability to administer PRN medications  - Gaining knowledge of any advance directive(s) related to PRN medications  - Association between PRN use, and workload and staff shortages  - Education of healthcare staff to comply with PRN medication standards  - Need for senior nurses to get involved in the PRN medication process  - Provision of instructions with enough detail for the appropriate use of PRN medications such as the dosage guideline  - Provision of training to healthcare providers in relation to PRN medications  - Consideration of the staffing pattern, shortages and inexperienced staff  - Nurses’ personal perspective and philosophy for PRN medication use  - Need for clear and up-to-date prescription information  - Appropriate storage of medications to facilitate access to medications  - Healthcare staff’s knowledge of medicines management  - Seeking for complementary competency through asking for the second opinion  - Practical knowledge and skills for the assessment of effects of PRN medications  - Appropriate staffing patterns in the ward  - Sharing verbal and written information  - Appropriate storage of medications to facilitate their access  - Culture of medication use as the use of non-pharmacological methods prior to medication use  - Presence of the individual medication protocol to decide on the administration of PRN medications  - Use of PRN medications based on the hospital’s protocol to prevent the use of restraints  - Use of PRN medications to manage sleep disturbances and help with adjustment to the work unit  - Provision of information in relation to patients’ medication during shift handoff  - Regulation of the use PRN medications in terms of reason for use, the schedule and route, the circumstances for use, the maximum dose, when to call the resident’s physician, and when to discontinue use  - Being ensured of patient safety in the caring environment | Healthcare professionals’ role |
| - Positive attitude by the patient and family members toward PRN medications use  - Patient’s preference and compliance with PRN medication use  - Expression of symptoms and request for medications by the patient  - Patient’s willingness and previous effectiveness to choose alternative methods  - Involvement of the patients for deciding on PRN medication use  - Patient’s knowledge of the list of medications  - Communication and cognitive abilities of patients to assess the necessity of PRN medication use  - Reaching agreements by the healthcare providers and families on PRN medications  - Being looked like unwell to receive PRN medications  - Association between the severity of experienced symptoms and dose of PRN medications  - Consideration of patient’s preferences for PRN use  - Interference of medications in the patient’s collaboration with the therapeutic plan  - Joint decision making about the prescription of medications wherever possible – including translating/agreeing the rational/indication for the prescription into the language of/with the service user  - Deconstructing instructions so that each action or intended behavior is separate and would potentially allow patients to be more cognizant of each step to be taken  - Deconstructing prescription wording for the core components of PRN instructions to explicitly convey the dose, interval between doses and the maximum daily dose  - Use of simplified text and plain language, e.g. “Stop” to replace the typical wording “Do not exceed” to convey the maximum daily dose among patients with limited literacy levels  - Patient’s request for medications or nurses’ decision making on PRN use;  - Use of the Take-Wait-Stop label design consisting of explicit, deconstructed instructions and simplified text: numeric characters instead of words, e.g., “1 tab” instead of “one tab” and “carriage returns” to place each part of the instructions on separate lines  - Use of word ‘stop’ instead of ‘do not exceed’ to convey the maximum daily dosage to patients in plain language  - Development of the Take‐Wait‐Stop Label, following the patient‐centered prescription label design  - Employing numeric characters instead of words, eg, “1 tab” instead of “one tab”, and “carriage returns” to place each section of the instruction on different lines | Participation of patients’ and families |
| - Discussing changes in PRN medication use between the physician and nurse  - Collaboration for the management of behaviors and reduction of medication use  - Creation of a consensus on PRN medication use through interprofessional medication review | Multidisciplinary collaboration |
